# Multiscale Frozen Density Embedding/Molecular Mechanics Approach for Simulating Magnetic Response Properties of Solvated Systems

**DOI:** 10.1021/acs.jctc.3c00850

**Published:** 2023-12-18

**Authors:** Piero Lafiosca, Federico Rossi, Franco Egidi, Tommaso Giovannini, Chiara Cappelli

**Affiliations:** † Scuola Normale Superiore, Piazza dei Cavalieri 7, 56126 Pisa, Italy; ‡ Software for Chemistry and Materials BV, De Boelelaan 1083, 1081 HV Amsterdam, The Netherlands

## Abstract

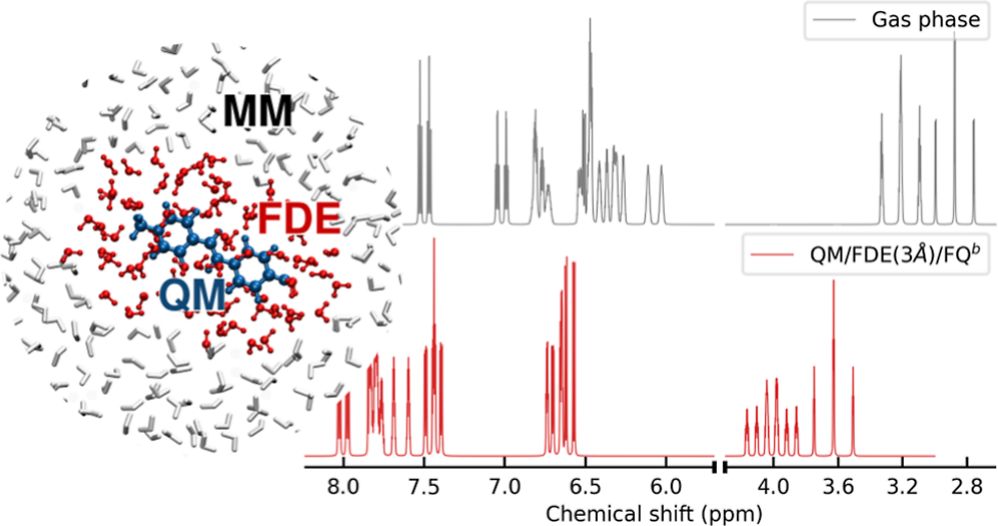

We present a three-layer hybrid quantum mechanical/quantum embedding/molecular mechanics approach for calculating nuclear magnetic resonance (NMR) shieldings and *J*-couplings of molecular systems in solution. The model is based on the frozen density embedding (FDE) and polarizable fluctuating charges (FQ) and fluctuating dipoles (FQFμ) force fields and permits the accurate ab initio description of short-range nonelectrostatic interactions by means of the FDE shell and cost-effective treatment of long-range electrostatic interactions through the polarizable force field FQ(Fμ). Our approach’s accuracy and potential are demonstrated by studying NMR spectra of Brooker’s merocyanine in aqueous and nonaqueous solutions.

## 1 Introduction

The simulation of molecular properties and spectra of complex molecular systems is challenging because the external environment can alter the electronic structure and, consequently, the electronic response of molecular systems. Under the assumption that the environment modifies, but does not determine, the molecular response of the embedded system, the so-called “focused models” have been developed and proven to yield reliable descriptions of experimental findings.^1−5^ There, the molecular space is partitioned into at least two layers, each treated at different levels of theory: the target, from which the signal originates, is usually described by means of accurate quantum mechanical (QM) methods, whereas the “environment” is treated at a lower level of accuracy. In this context, QM/classical methods have emerged as the most successful for the description of large embedded systems thanks to the reduced computational cost associated with the classical portion, which can be treated either as a continuum dielectric or in a fully atomistic way.^6,7^ The latter case, i.e., the so-called QM/molecular mechanics (QM/MM) approach, is generally preferable for strongly interacting molecule-environment systems, for instance, in the case of solutions that are dominated by directional and specific hydrogen-bonding (HB) interactions (e.g., aqueous solutions).

QM/MM approaches generally treat the interaction between the two layers (classical and QM) at the purely electrostatic level; mutual polarization effects can indeed be included, giving rise to the so-called polarizable embedding methods, which yield a more physically consistent picture of the chemical system.^4,8−12^ In most QM/classical methods, nonelectrostatic effects between the QM and classical portions, such as Pauli repulsion and dispersion, are neglected. However, these interactions can play an essential role in many systems, ranging from solutions^13,14^ to biosystems.^15−17^ Effective methods to introduce these interactions in QM/MM methods have been proposed, but their accuracy crucially relies on the appropriateness of parametrization.^18^ An alternative approach is to resort to quantum embedding methodologies,^19−40^ which permit a correct description of Pauli repulsion by ensuring the orthogonality between the molecular orbitals of the two regions. However, such techniques are generally much more computationally expensive compared to QM/classical approaches.

A possible compromise between QM/classical and quantum embedding methods has been proposed recently by some of us.^41^ In our three-layer model, the inner core is treated with a high-level QM method, the intermediate layer is modeled by means of the frozen density embedding (FDE) method,^36,42−49^ and the outer shell is described at the classical level by using the fluctuating charges force-field (FQ).^4^ The resulting QM/FDE/FQ model takes into account short-range and nonelectrostatic interactions by means of the FDE shell and retains long-range, electrostatic–polarization interactions, by means of the FQ layer, in a cost-effective way. The description of the outer FQ shell can be further refined by adding a set of fluctuating dipoles (QM/FQFμ),^50−52^ which permit a more sophisticated description of long-range electrostatic interactions, thanks to the inclusion of anisotropic polarization sources.

In this work, we extend QM/FDE/FQ to the simulation of nuclear magnetic resonance (NMR) shielding and spin–spin *J* coupling constants. NMR shielding is a near-sighted property; in fact, the chemical environment around each nucleus plays a key role in the local magnetic field and consequently in the shielding value. For these reasons, it is considered one of the most challenging properties for solvation models.^53^

The paper is organized as follows. In the next section, QM/FDE/FQ and QM/FDE/FQFμ are outlined and extended to compute NMR shieldings and spin–spin *J* coupling constants. After a short description of the technical details of the computational methodology, QM/FDE/FQ(Fμ) is challenged to compute NMR spectra of Brooker’s merocyanine (MOED)^54^ as dissolved in aqueous and nonaqueous solutions.

## 2 Theory

### 2.1 QM/FDE/FQ and QM/FDE/FQFμ Models

The total energy functional of the three-layer QM/FDE/FQ(Fμ) model can be written as^41^
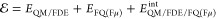
1

The first term in eq 1 represents the energy of the QM/FDE portion,^55^ which is constituted by two subsystems I and II. In this region, the total electron density ρ_tot_(**r**) is given by the sum of the densities ρ_I_(**r**) and ρ_II_(**r**) of the subsystems, i.e.
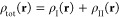
2

The two densities are allowed to overlap and are required to integrate into an integer number of electrons. The total energy functional can be written as a bifunctional of the two densities, where the kinetic term is calculated by using Kohn–Sham (KS) orbitals of the noninteracting reference system for each subsystem.^55^ The total energy bifunctional can then be expressed as follows
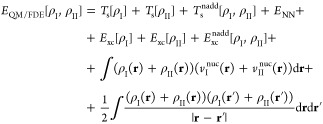
3where *E*_NN_ is the nuclear repulsion energy, *v*_I_^nuc^ and *v*_II_^nuc^ are the nuclear electrostatic potentials of subsystems I and II, and *E*_xc_ is the exchange–correlation (xc) energy functional, which is decomposed as the sum of the xc-functionals for each density and a nonadditive term *E*_xc_^nadd^ due to the nonlinearity of *E*_xc_. A nonadditive kinetic energy term *T*_s_^nadd^[ρ_I_,ρ_II_] = *T*_s_[ρ_I_ + ρ_II_] – *T*_s_[ρ_I_] – *T*_s_[ρ_II_] is also included in order to account for the full kinetic energy; however, in practical implementations, this term is treated by means of approximated functionals.^55^

The second term in eq 1, *E*_FQ(Fμ)_, is the energy of the classical region. Within the FQ force field,^4^ a charge *q* is assigned to each atom; remarkably, charge values are not fixed but are determined from the polarization response of the atom to the environment, according to the electronegativity equalization principle.^56,57^ The electrostatic description provided by the FQ model can be enriched by introducing atomic dipoles, leading to the fluctuating charges and fluctuating dipoles (FQFμ) force-field.^50−52^ In this method, an additional electric dipole **μ** free to fluctuate as a response to external perturbation is located on each atom of the environment, thus representing anisotropic interactions. The energy functional for the FQ(Fμ) force field can be written in a compact formulation as
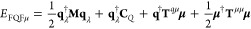
4where the first two terms correspond to the FQ force field energy.^4^ In particular, **q**_λ_ is a vector containing FQ charges **q** and a set of Lagrange multipliers **λ**, which are introduced to fix the total charge *Q* on each FQ molecule, thus avoiding intermolecular charge transfer. **M** is a matrix collecting the charge–charge interaction kernel (**T**^*qq*^) and a set of Lagrangian blocks.^58^**T**^*qq*^ diagonal elements describe the charge self-interaction energy and are expressed in terms of atomic chemical hardnesses η. **C**_*Q*_ is a vector containing atomic electronegativities **χ** and charge constraints **Q**.

The last two terms in eq 4 specify the FQFμ force field and represent the charge–dipole and dipole–dipole interactions, which are mediated by the **T**^*q*μ^ and **T**^μμ^ interaction kernels. In particular, the dipolar self-interaction, i.e., the diagonal elements of the **T**^μμ^ tensor, are expressed in terms of the atomic polarizabilities α.

The last term in eq 1, *E*_QM/FDE/FQ(Fμ)_^int^, is the interaction energy between the quantum (QM/FDE) and classical (FQ or FQFμ) portions. It is expressed as the electrostatic interaction between the multipolar moments of the environment and the total electric potential and field generated by the QM/FDE subsystems. By exploiting the definition of ρ_tot_ given in eq 2 and the linearity of the electrostatic potential with respect to the electric sources, *E*_QM/FDE/FQ(Fμ)_^int^ can be written as follows
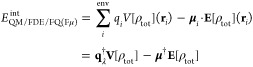
5where *i* runs over the atoms of the environment (env). *V*[ρ_tot_](**r**_*i*_) and **E**[ρ_tot_](**r**_*i*_) are the QM potential and field evaluated at position **r**_*i*_ of the *i*-th atomic site in the environment, i.e.
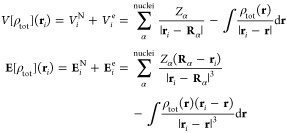
6where the potential and field are partitioned into nuclear (*V*_*i*_^N^, **E**_*i*_^N^) and electronic terms (*V*_*i*_^e^, **E**_*i*_^e^). *Z*_α_ indicates nuclear charges of QM/FDE atoms, each located at positions **R**_α_.

By collecting all terms together, eq 1 can be rewritten as
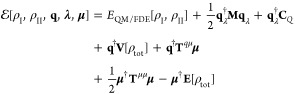
7

Notice that QM/FDE/FQ is obtained by discarding the last three terms in eq 7 (i.e., by retaining the terms exclusively depending on charges).^41^ Given the total energy functional, the working equations to determine the densities of the QM/FDE portion and the polarizable multipoles of the environment can be obtained by a constrained minimization of eq 7 with respect to charges (dipoles) and Lagrange multipliers. This leads to the following linear system where the second term on the right-hand side describes the mutual polarization between the QM/FDE and FQ(Fμ) regions.


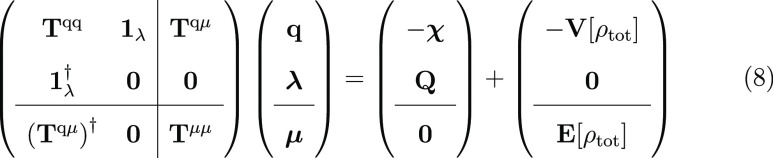
8

To obtain the self-consistent field procedure for the QM/FDE scheme, ρ_II_ (the density describing the environment) is kept frozen, while ρ_I_ is determined by minimizing the energy functional with respect to the density itself, under the constraint that the number of electrons *N*_I_ is conserved. The minimization of the energy functional in eq 7 with respect to ρ_I_ reads
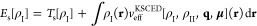
9where *v*_eff_^KSCED^ is the effective potential for the noninteracting reference system, i.e.
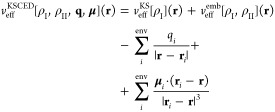
10

In the previous equation, *v*_eff_^KS^ is the KS effective potential of subsystem I and *v*_eff_^emb^[ρ_I_,ρ_II_] describes the interaction of subsystem I with the frozen density and the nuclear charges of subsystem II. The last two terms represent the modification of the KS effective potential as obtained by introducing the FQ(Fμ) shell.

The above formulation of QM/FDE does not account for the mutual polarization between the two subsystems. The model can be improved by using the so-called freeze-and-thaw iterations,^59^ in which the role of the frozen and the nonfrozen subsystems is alternatively exchanged in order to take into account the effect of the relaxation of each density on that of the other subsystem. Such an approach is generally referred to as “subsystem DFT”.^60,61^

### 2.2 Nuclear Magnetic Resonance Shielding and Spin–Spin Coupling Constants for QM/FDE/FQ and QM/FDE/FQFμ Models

QM/FDE/FQ has been extended to the calculation of excitation energies of embedded molecules in a time-dependent density functional theory framework in ref (41). A similar derivation can be extended to QM/FDE/FQFμ. In this work, QM/FDE/FQ(Fμ) is extended to simulate the NMR spectra of solvated systems.

NMR is one of the most powerful and applied spectroscopic techniques in chemistry for the determination of the molecular structure and the spatial arrangement of functional groups. The sample, usually a solution, is placed in an external magnetic field, and the local magnetic fields around atomic nuclei are probed by excitation with radio waves. Quantum-chemical calculations can support the interpretation of NMR spectra by providing the chemical shielding tensor **σ**_α_, which describes the relative change in the local magnetic field on the atom α. NMR experimental measurements are generally reported in terms of the chemical shift **δ**_α_, which is the variation of the resonance frequency with respect to a reference compound. The relation between **σ**_α_ and **δ**_α_ is given by^62^
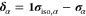
11where σ_iso,α_ is the isotropic value of the chemical shielding tensor of the standard reference of the same atom and **1** is the 3 × 3 identity matrix.

NMR shielding **σ**_α_ tensor components are defined as the second derivative of the total electronic energy  with respect to a constant external magnetic field **B** and the nuclear magnetic moment **μ**_α_,^63^ i.e.
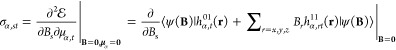
12where the indices *r*, *s*, *t* run over the Cartesian directions. The expression is obtained by exploiting double perturbation theory^64^ and depends on ψ(**B**), which is the ground-state electronic eigenfunction under the influence of the external magnetic field. The operators *h*^01^ and *h*^11^ are given by^65^
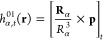
13
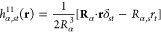
14where **r** and **p** are the electronic position and momentum operators and **R**_α_ is the position of the nucleus α. The operator in eq 14 is bilinear in **B**_α_ and **μ**_α_. Thus, it yields the diamagnetic shielding, while *h*^01^ is linked to paramagnetic shielding.^66^

Notice that eq 3 should be modified to include the external magnetic field, which can be done at the nonrelativistic level by replacing **p** with , where **A** is the magnetic vector potential.^67^ The exchange–correlation potential should also include a dependence on the current density but this is usually neglected.^68^ It is also worth noting that in order to avoid the dependence of the result on the choice of the gauge for the vector potential **A**, especially due to the selection of the coordinate origin, “gauge-including atomic orbitals” are exploited.^69^

Nuclear spin–spin coupling constants **K** describe the interaction between two nuclear magnetic moments. They can be defined as the second derivative of the total electronic energy  with respect to the nuclear magnetic moments **μ**_α_ and **μ**_β_ of the involved nuclei^70^
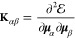
15

This quantity can be related to the ordinary spin–spin coupling constant **J** through the expression
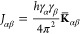
16where  is the isotropic part of **K**_αβ_, usually called “reduced spin–spin coupling constant”,^70^ γ_α_, γ_β_ are the nuclear magnetogyric ratios, and *h* is the Planck constant. The evaluation of eq 15 for a nonrelativistic Hamiltonian leads to a modification of the KS operator with four additional terms^71^
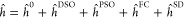
17

The second term is the diamagnetic spin–orbit (DSO) operator, which is the only one to be bilinear in the two magnetic moments and thus gives a contribution that only depends on the unperturbed KS orbitals. Its definition reads
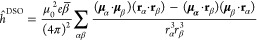
18where μ_0_ is the permeability of the vacuum, β̅ is the Bohr magneton, and **r**_α_, **r**_β_ are the position vectors of the electron with respect to the nuclei α and β, respectively. The third term in eq 17 is the paramagnetic spin–orbit (PSO) operator, which is defined as
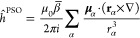
19

The Fermi Contact (FC) operator instead reads
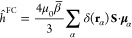
20where δ is the Dirac delta function and **S** is the spin momentum operator. Finally, the last term in eq 17 is the spin-dipolar (SD) operator
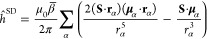
21

These operators represent different contributions to **K**_αβ_, which are determined by solving a set of coupled perturbed equations. As for NMR shieldings, the dependence of the exchange–correlation potential on the current density is neglected.^72^

The QM/FDE scheme has been extended to the calculation of chemical shieldings and spin–spin coupling constants.^43,73,74^ Also in this case, the dependence of the exchange–correlation potential and the nonadditive kinetic terms on the current density is neglected, as it is usual for DFT NMR calculations. As a consequence, the chemical shieldings in eq 12 can be calculated independently for the active and frozen subsystems and then simply added together.^73^ The computation of the spin–spin coupling constants requires the evaluation of the four terms in eq 17, and if we are only interested in the spin–spin coupling tensors of the active subsystem, a very efficient computational scheme can be obtained (see ref (74)).

In the case of QM/FDE/FQ and QM/FDE/FQFμ methods, the inclusion of the outer FQ(Fμ) shell is not associated with explicit contributions because the related quantities do not depend on the nuclear magnetic moment.^75^ Since the effect of the polarizable embedding is only implicit in the modification of the KS orbitals of the target system, the chemical shielding tensor and the spin–spin coupling constants can be obtained directly from the evaluation of eqs 12 and 15.

## 3 Computational Details

**Figure 1:**
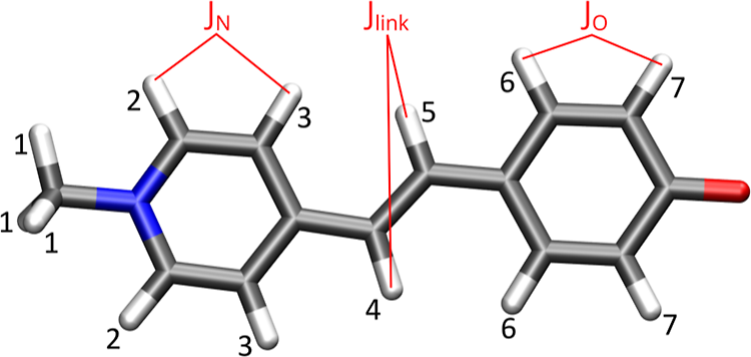
Molecular structure of MOED. In the following, we will adopt a notation for the group of equivalent protons: (1) methyl; (2) N-ortho; (3) N-meta; (4) N-link; (5) O-link; (6) O-meta; (7) O-ortho. The spin–spin coupling constants between adjacent protons have also been labeled as *J*_N_ (N-ortho and N-meta), *J*_link_ (N-link and O-link), and *J*_O_ (O-ortho and O-meta).

In this work, we apply QM/FDE/FQ and QM/FDE/FQFμ to calculate NMR chemical shieldings and spin–spin coupling constants of MOED^54^ (see Figure 1) as dissolved in water, ethanol, acetonitrile, and tetrahydrofuran (THF).

**Figure 2:**
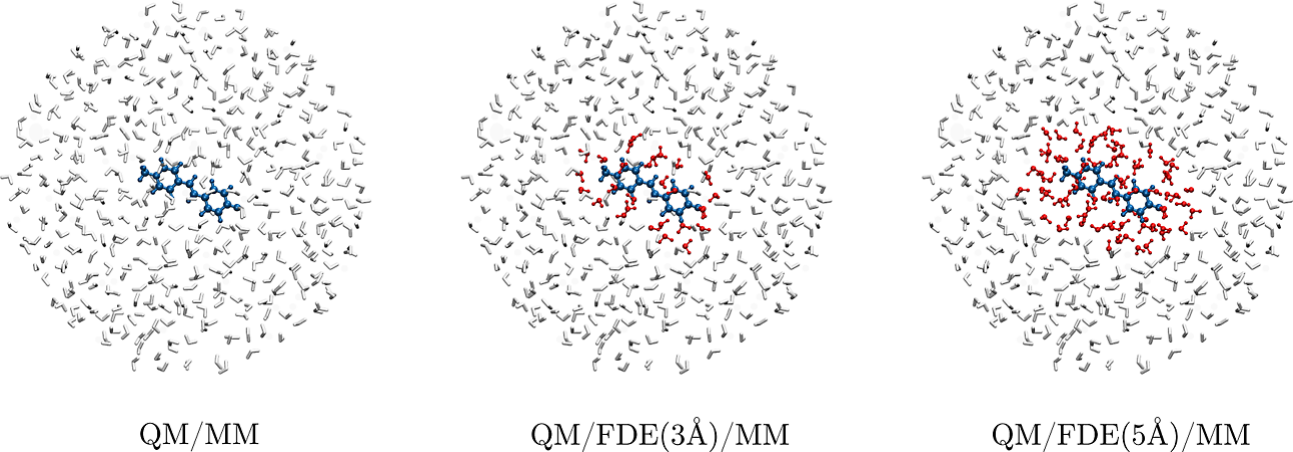
Pictorial view of the three approaches that are applied to the modeling of the solvated MOED.

The first step of our computational protocol is the conformational sampling of the system by resorting to a classical molecular dynamics (MD) simulation in order to take into account the dynamics of the solvent around the solute. In a previous work of some of the authors, several MD runs were performed on the MOED molecule in solution (for more technical details, see ref (76)). By following this study, MD calculations were performed in GROMACS^77^ and intramolecular and intermolecular interactions are described by means of the general AMBER force field.^78,79^ All bonded and nonbonded parameters for the solute and solvent are generated with the Antechamber package,^80,81^ while for the case of the aqueous solution, the solvent is treated by means of the standard TIP3P force field.^82^ During each MD simulation, MOED geometry is kept fixed in its minimum energy structure computed at the CAM-B3LYP/aug-cc-pVDZ level of theory, where solvent effects are included by means of the polarizable continuum model method.^1^ Each conformational sampling is obtained by following a two-step protocol: first, a 1 ns equilibration step in the *NPT* ensemble, by using the Berendsen barostat^83^ and a coupling constant of 2.0 ps; after that, a 2.5 ns *NVT* production run to sample the solvent configuration space around the fixed MOED. In both cases, a common integration time step of 1 fs is chosen. The temperature is kept fixed to 300 K by adopting the velocity-rescaling method^84^ with a coupling constant of 0.1 ps. As the last step of the conformational sampling, 200 uncorrelated snapshots are extracted from the last 2 ns of the *NVT* MD run, and a sphere of 22 Å of radius centered in the MOED center of mass is cut. The resulting spherical structures are employed in NMR calculations. In the latter, the solute is treated at the QM level, whereas the solvent molecules within a sphere of 22 Å of radius are modeled by exploiting three different approaches (see also Figure 2):

1. QM/MM: all solvent molecules are treated with a MM force field, either FQ or FQFμ (see Figure 2a).
2. QM/FDE(3 Å)/MM: the solvent molecules with at least one atom within 3 Å from at least one of the solute atoms are treated as frozen in the FDE layer, whereas all the remaining solvent molecules are described at the MM (FQ or FQFμ) level (see Figure 2b).
3. QM/FDE(5 Å)/MM: the solvent molecules with at least one atom within 5 Å from at least one of the solute atoms are treated as frozen in the FDE layer, whereas all the remaining solvent molecules are described at the MM (FQ or FQFμ) level (see Figure 2c)

In all calculations, MOED is described by exploiting the hybrid B3LYP exchange–correlation functional in combination with the DZP basis set.^85^ In the case of QM/FDE(3 Å)/MM and QM/FDE(5 Å)/MM calculations, as a preliminary step, the frozen density of the FDE layer is computed. This is obtained by performing a QM/MM single point calculation at the B3LYP/DZP level in which the QM layer consists only of the solvent molecules in the FDE layer, while the other solvent molecules are included in the MM shell (see also ref (41)). The converged ground-state density is then kept frozen in the subsequent QM/FDE/MM calculation. To calculate FDE terms, the PW91K^86^ functional has been chosen to approximate the kinetic energy, whereas the nonadditive exchange–correlation terms are treated by means of the PBE functional.^45^ The choice of the PW91K follows earlier studies on the simulation of ground-state properties^87,88^ and NMR spectra^46,73,74^ by using FDE. In the case of aqueous solutions, three different MM models are exploited in NMR calculations: TIP3P, a fixed charges embedding approach;^82^ FQ, for which two different parametrizations are taken into account, namely, FQ^a^ (from Rick and co-workers, see ref (89)) and FQ^b^ (from ref (76)); FQFμ, with the parameters reported in ref (50). Nonaqueous solvents are treated only with the FQ^b^ method, with the parameters reported in ref (76). As described above, FQ and FQFμ require the definition of the off-diagonal elements of the interaction tensors **T**^*qq*^, **T**^*q*μ^, and **T**^μμ^ in eq 4. In the case of FQ calculations, Ohno’s kernel is adopted,^90^ whereas in the FQFμ method, the interaction tensors are obtained by assuming a Gaussian distribution of the multipolar moments, leading to the expressions reported in ref (50).

Ground-state calculations are performed in a locally modified version of the ADF program,^91^ from the Amsterdam Modeling Suite (AMS) software package.^92^ The computation of the ground-state density in ADF is performed through a numerical integration scheme; therefore, the electric potential and field generated on the MM portion are evaluated numerically (see eq 6). In order to avoid numerical instabilities, QM/MM coupling terms have been modified by including a screening function of the interatomic distance.^93^

The computation of chemical shieldings and spin–spin *J* coupling constants are performed by means of the NMR and CPL packages, respectively, which are part of the AMS suite of programs.^63,74^ The internal reference for both ^1^H NMR and ^13^C NMR spectra is tetramethylsilane (TMS), with a value of the shielding set to 31.7 and 182.852 au, respectively. To generate NMR spectra, chemical shift and *J* are averaged among all 200 snapshots, which guarantee the convergence of the results (see Figures S2 and S3 in the Supporting Information). All the averaged couplings between protons with *J* ≥ 0.5 Hz are considered and converted to parts per million, assuming a magnetic field of 400 MHz. The results are then convoluted using Lorentzian functions with the full width at half-maximum equal to 0.002 ppm.

Cartesian coordinates of MOED in the gas phase and a summary of the keywords adopted in our calculations are reported in Section S1 of the Supporting Information.

## 4 Numerical Applications

In this section, we first apply QM/FDE/MM to the calculation of the NMR spectra of MOED dissolved in water. In particular, we assess the quality of our approach by varying the tunable parameters defining the method, i.e., the size of the FDE shell, and the effect of polarization contributions. Various MM approaches, ranging from nonpolarizable MM to polarizable FQFμ, are tested, and computed values are compared with experimental data recovered from the literature.^94^ Finally, to demonstrate the reliability and robustness of the approach, NMR parameters are computed for MOED in nonaqueous solutions.

### 4.1 Dependence of the Results on the Size of the FDE Shell

We first focus on the effect on computed NMR shielding of varying the size of the FDE shell. This is indeed a relevant tunable (and arbitrary) parameter in QM/FDE/MM approaches, which however crucially determines the region, giving rise to nonelectrostatic effects on the QM target.

**Figure 3:**
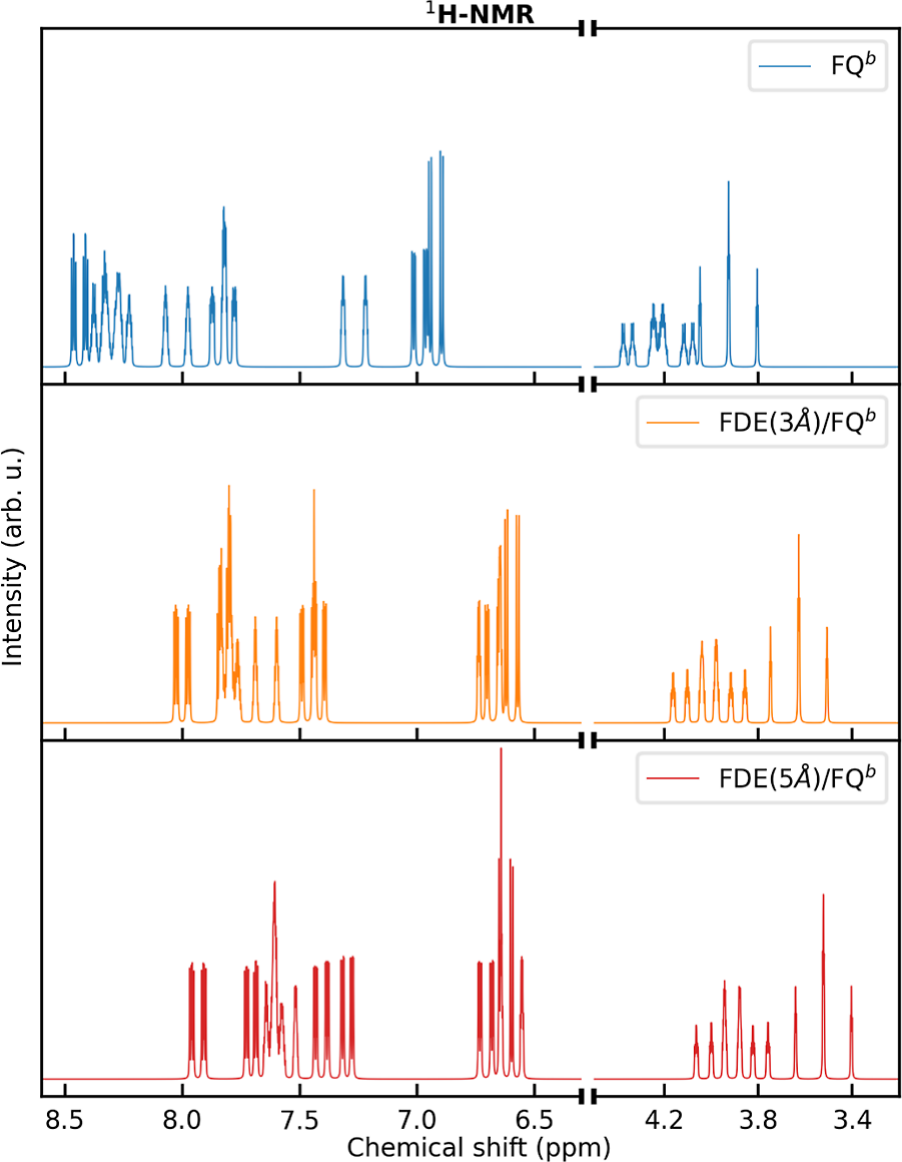
^1^H NMR spectra of MOED in aqueous solution, as computed at the QM/FQ^b^ (top panel), QM/FDE(3 Å)/FQ^b^ (middle panel), and QM/FDE(5 Å)/FQ^b^ (bottom panel) levels.

In Figure 3, we report MOED ^1^H NMR spectra in aqueous solution at the QM/FQ^b^ and QM/FDE/FQ^b^ levels. The FDE shell has dimensions 3 Å (QM/FDE(3 Å)/FQ^b^) and 5 Å (QM/FDE(5 Å)/FQ^b^). All spectra are characterized by three separate regions: (i) 3 < ppm <4.5 related to methyl hydrogen atoms; (ii) 6.5 < ppm <7 (QM/FDE/FQ^b^) and 6.5 < ppm <7.5 (QM/FQ^b^) related to N-link and O-ortho hydrogen atoms; (iii) ppm >7 (QM/FDE/FQ^b^) and ppm >7.5 (QM/FQ^b^) related to N-ortho, O-meta, N-meta, and O-link hydrogen atoms. As can be noticed, the two quantum embedding methods (QM/FDE(3 Å)/FQ^b^ and QM/FDE(5 Å)/FQ^b^) provide very similar results, with minor discrepancies reported for methyl protons (see Tables S2–S5 in the Supporting Information). This can probably be related to the fact that methyl protons are more exposed to solvent molecules. Therefore, the convergence of short-range nonelectrostatic contributions as a function of the FDE shell is slower with respect to other atoms. However, the effect of increasing the FDE shell from 3 to 5 Å is remarkably negligible, in particular, when compared with the QM/FQ^b 1^H NMR spectrum reported in Figure 3. For this reason, in the following, we focus on the QM/FDE(3 Å)/MM (named QM/FDE/MM) partition only, as the best compromise between computational cost and accuracy.

**Figure 4:**
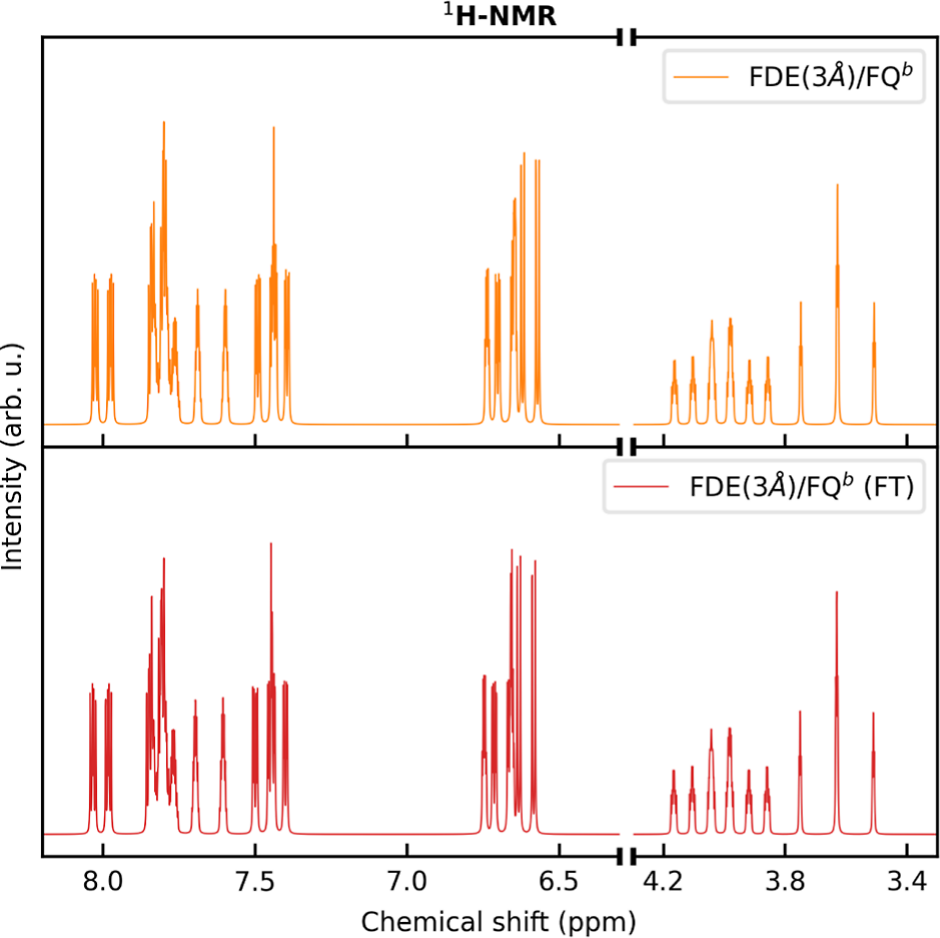
^1^H NMR spectra of MOED in aqueous solution as computed at the QM/FDE(3 Å)/FQ^b^ level by using unrelaxed (top panel) and relaxed (freeze-and-thaw, FT, bottom panel) densities for the FDE shell.

As stated above, the FDE layer is described by means of a frozen density obtained independently of that of the solute. This approximation can be refined by exploiting the so-called subsystem DFT approach.^59^ This consists of performing a set of “freeze-and-thaw” (FT) cycles in which the roles of the solute and environment densities switch, leading to a more accurate description of mutual polarization between the two layers. In Figure 4, we report MOED ^1^H NMR spectra in an aqueous solution as computed at the QM/FDE/FQ^b^ level by considering a frozen and a relaxed FDE shell (FT). The two spectra are almost identical. Indeed, the discrepancies are limited to a slight change in relative intensities, while both the chemical shifts and the spin–spin coupling constants are almost unaffected by the FT procedure (see also Tables S2, S4, and S6 in the Supporting Information). This is not surprising and it is in agreement with previous works,^95,96^ which highlighted that the relaxation of the environment density in the case of neutral embedded species does not lead to improvement of computed properties. Note that our findings are also in agreement with our previous work on absorption spectra computed at the QM/FDE/FQ level,^41^ where we showed that the relaxation of the FDE density does not substantially affect computed excitation energies. Based on these results, in the following, we focus on the common FDE formulation, without resorting to FT optimization, leading to a huge reduction in computational cost.

### 4.2 NMR Spectra Obtained by Changing the Embedding Model

**Figure 5:**
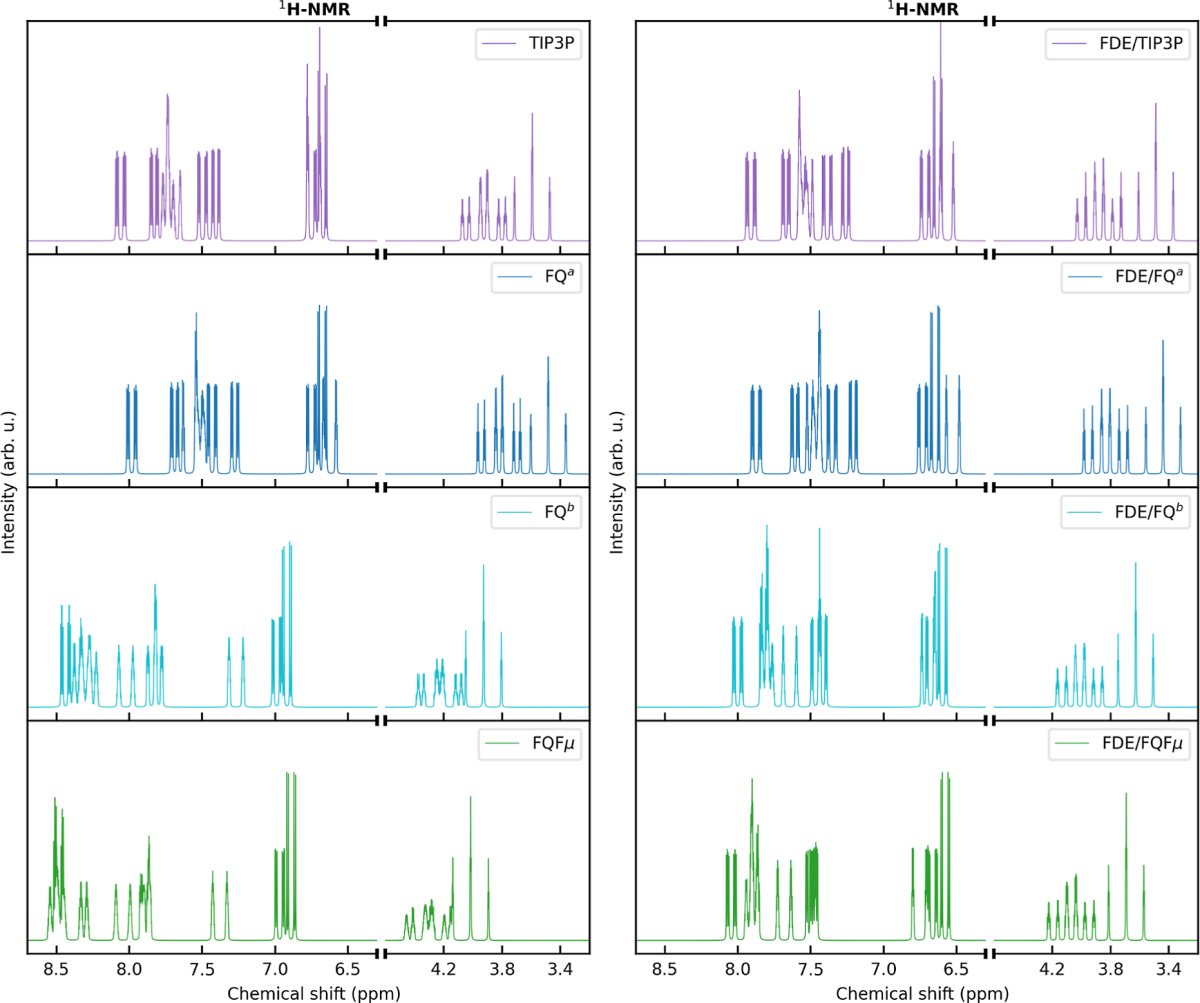
^1^H NMR spectra of MOED in aqueous solution computed at the QM/TIP3P, QM/FQ^a^, QM/FQ^b^, and QM/FQFμ levels (left panel) and QM/FDE(3 Å)/TIP3P, QM/FDE(3 Å)/FQ^a^, QM/FDE(3 Å)/FQ^b^, and QM/FDE(3 Å)/FQFμ (right panel) levels.

In this section, we investigate the dependence of ^1^H NMR spectra on the choice of the embedding model. In particular, we start from the nonpolarizable QM/TIP3P and polarizable QM/FQ^a^, QM/FQ^b^, and QM/FQFμ (see Figure 5a), and we add an intermediate FDE layer (QM/FDE/TIP3P, QM/FDE/FQ^a^, QM/FDE/FQ^b^, and QM/FDE/FQFμ—see Figure 5b). Let us first focus on the QM/MM results (Figure 5a). All spectra are characterized by the three aforementioned separate regions, with the same assignment outlined above. As can be noticed from the inspection of the chemical shifts, both QM/TIP3P and QM/FQ^a^ predict overall larger proton shielding with respect to QM/FQ^b^ and QM/FQFμ. Such a discrepancy can be explained by considering that TIP3P and FQ^a^ force fields are parametrized to reproduce the energy and properties of bulk liquid water,^82,89^ while the parameters defining FQ^b^ and FQFμ force fields are obtained so as to reproduce the electrostatic (and polarization) components of the interaction energy between water molecules.^50,76^ As a consequence, the attractive electrostatic and polarization energy terms in both FQ^b^ and FQFμ are expected to be larger than those calculated by means of FQ^a^ and TIP3P. Indeed, the inclusion of solute–solvent electrostatic (and polarization) interactions, as for any of the QM/MM approaches, induces a delocalization of MOED electronic density toward the solvent molecules. As a consequence, MOED protons are likely to be less shielded with respect to the gas-phase reference, leading to larger chemical shifts (see also Table S7 in the Supporting Information). The above considerations are also confirmed by noting that the shifts computed at QM/FQ^b^ and QM/FQFμ levels with respect to the gas-phase reference are more pronounced than those calculated by exploiting QM/TIP3P and QM/FQ^a^ (see Table S7 in the Supporting Information).

We now move to the MOED ^1^H NMR computed spectra at the QM/FDE(3 Å)/MM level, which are graphically depicted in Figure 5b. At first glance, by comparison of Figure 5a,b, the presence of the FDE shell induces an overall shielding of the protons for all the MM models, leading to a reduction of the chemical shift. Such an effect is associated with the inclusion of nonelectrostatic contributions between the QM and the FDE subsystems. Short-range interactions, which follow from the Pauli principle, lead to an overall confinement of the electron density on MOED nuclei. From an inspection of the numerical values (see Table S7 in the Supporting Information), it can be noticed that the effect of the inclusion of the FDE shell for each MM embedding model is almost uniform across all the protons and generally yields a reduction of the chemical shift (negative shift). In this case, the shielding is largest for the QM/FDE/FQ^b^ and QM/FDE/FQFμ models, for which their QM/MM counterparts are characterized by the largest chemical shifts. However, it is worth remarking that all ^1^H NMR spectra computed by including the FDE shell present only minor discrepancies upon changing the MM force field. This is due to the similar description of short-range interactions (the FDE layer).

### 4.3 Role of the Solvation Shells

NMR shielding is a near-sighted property determined by the screening of induced currents (by external or internal magnetic fields) close to the atomic nuclei. Therefore, it is worth separating and investigating the effect on NMR spectra of the FDE and FQ regions in our composite QM/FDE/FQ scheme. In particular, the inclusion of the outer FQ shell is associated with both explicit and implicit effects. The explicit effect is the electrostatic interaction term entering eq 10, which has a direct effect on the determination of the ground-state density. The implicit effect is instead associated with the procedure exploited to determine the FDE density. As explained in the computational details, in a QM/FDE/FQ calculation, the FDE density is obtained via a ground-state QM/FQ calculation in which the QM region is composed of the water molecules included in the FDE layer, while the other solvent molecules are treated at the FQ level. Within this procedure, the FDE density is polarized by the outer FQ shell (implicit effect).

**Figure 6:**
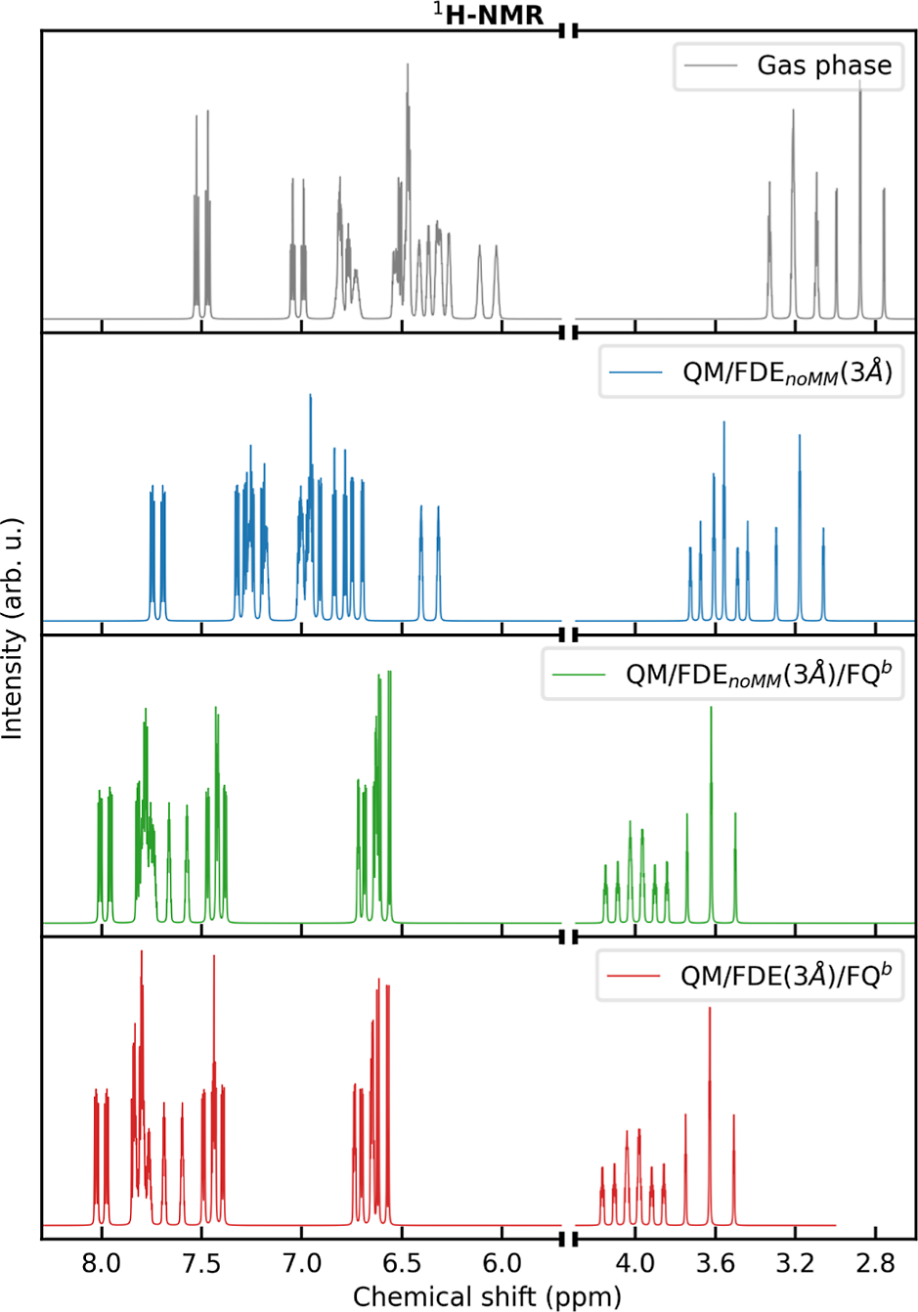
^1^H NMR spectra of MOED in the gas phase (top panel) and aqueous solution, as computed at the QM/FDE_noMM_(3 Å) (middle top panel), QM/FDE_noMM_(3 Å)/FQ^b^ (middle bottom panel), and QM/FDE(3 Å)/FQ^b^ (bottom panel) levels.

To evaluate the effect of the outer MM layer and to dissect the two associated contributions, we have computed ^1^H NMR spectra for the following cases: gas-phase MOED; QM/FDE_noMM_(3 Å), in which the outer MM layer is discarded; QM/FDE_noMM_(3 Å)/FQ^b^, in which only the explicit effect of FQ^b^ is taken into account (i.e., the FDE density is obtained without considering the external MM layer); QM/FDE(3 Å)/FQ^b^, in which both explicit and implicit effects are included. The obtained results are listed in Figure 6.

As stated above, the MOED geometry is kept frozen during the MD simulation; thus, MOED protons are not equivalent because they experience different chemical surroundings. This is not a solvent-induced effect since this feature is also present in the gas phase (gray line in Figure 6). As an example, the signals of the methyl hydrogen appear in the region between 3.4 and 2.6 ppm as a pair of triplets because the methyl hydrogen lying on the MOED plane (see Figure 1) has a chemical shielding which is different from that of the other two hydrogen atoms (see raw data in Table S7 in the Supporting Information). Remarkably, the set of protons that are most affected by the lack of equivalence is those of the N-meta, O-meta, and methyl groups (see Figure S7 in the Supporting Information). A similar effect is reported also for the coupling constants (see raw data in Table S8 in the Supporting Information).

**Figure 7:**
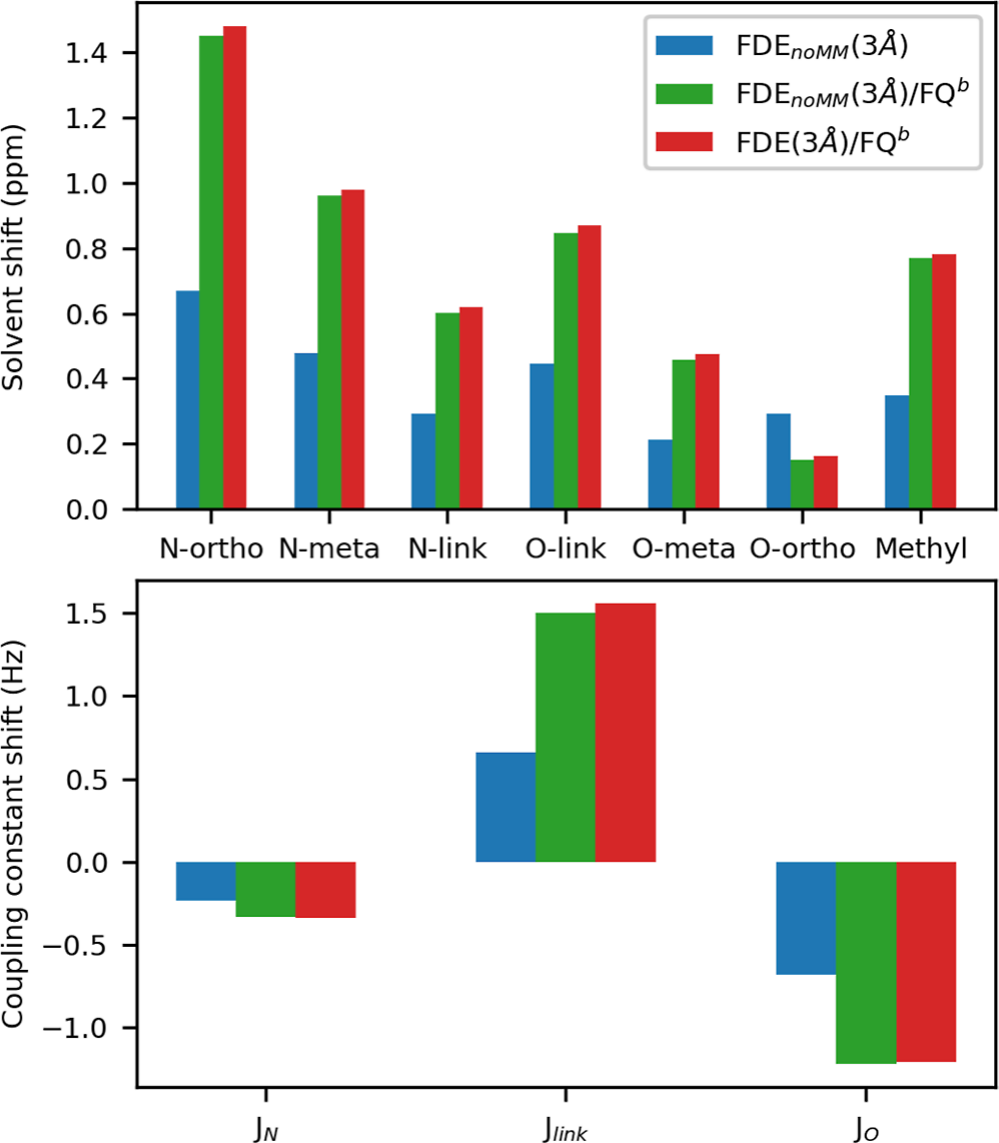
QM/FDE_noMM_(3 Å), and QM/FDE_noMM_/FQ^b^, QM/FQ^b^ vacuo-to-aqueous solution variations in the chemical shift (ppm, upper panel) and *J* coupling constants (Hz, bottom panel).

Including the aqueous environment leads to a general deshielding of all protons, which can be associated with the spill out of the MOED density toward the solvent region compared to the vacuum. To quantify this effect, in Figure 7, we report vacuo-to-solvent shifts of chemical shifts and coupling constants. As it can be appreciated, including the outer FQ^b^ shell has a relevant impact on the hydrogen shifts and coupling constants, almost doubling or halving the values which are obtained by only including the FDE_noMM_ layer (see also Figure S4 in the Supporting Information). Remarkably, this effect is almost entirely determined by the explicit FQ contribution because QM/FDE_noMM_(3 Å)/FQ^b^ and QM/FDE(3 Å)/FQ^b^ spectra are almost identical (see Table S13 in the Supporting Information for the raw data).

### 4.4 Comparison with Experimental Data

We now move on to comparing the computed ^1^H NMR spectra of MOED in an aqueous solution with experimental data reported for MOED in deuterated water in ref (94). In order to compare our data with the experiment, the chemical shifts and the coupling constants associated with equivalent protons are averaged (see Figure 1).

**Figure 8:**
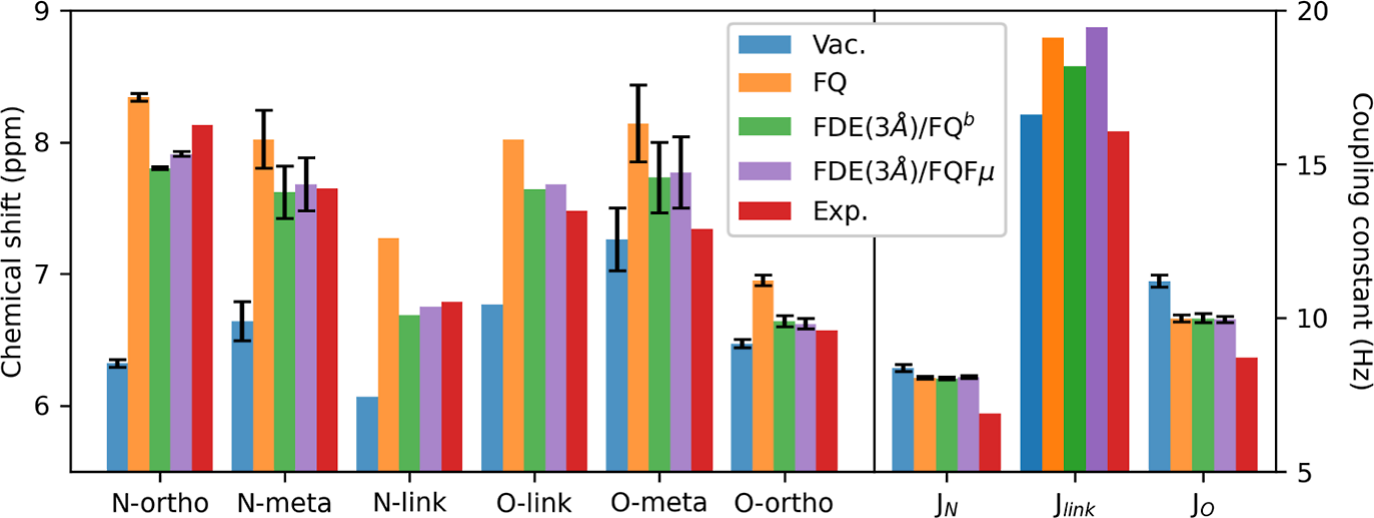
Computed QM/FQ^b^, QM/FDE(3 Å)/FQ^b^, and QM/FDE(3 Å)/FQFμ ^1^H NMR chemical shifts (ppm) and *J* coupling constants (Hz) of MOED dissolved in aqueous solution. Gas-phase (vac) and experimental data (exp) are also reported.

Computed gas-phase, QM/FQ^b^, and QM/FDE/FQ^b^ chemical shifts and spin–spin coupling constants of MOED in water are reported in Figure 8, together with their experimental counterparts (see Table S14 in the Supporting Information for the raw data).^94^ Notice that for equivalent hydrogen atoms (N-ortho, N-meta, O-meta, and O-ortho), the half-differences are also reported (in parentheses). QM/FQ^b^ and QM/FDE/FQ^b^ report an increase with respect to their gas-phase reference for all considered proton chemical shifts. In particular, for all protons, the results for the isolated molecule are smaller than experimental values, whereas a much better agreement is found in the case of the solvated system. However, QM/FQ^b^ tends to overestimate all chemical shifts, while the inclusion of the FDE shell reduces all computed chemical shifts, thus leading to a generally better agreement with the experimental data. This can be rationalized by considering that QM/FQ^b^ accounts for only purely electrostatic and polarization interactions, which induce a larger deshielding by reducing the electron density on the nuclei. Differently, the much better agreement obtained using QM/FDE/FQ^b^ demonstrates the crucial effect of nonelectrostatic contributions, particularly of Pauli repulsion. It is however worth noting that the only significant discrepancy with the experiment is reported for meta hydrogens (N-meta and O-meta), for which an opposite behavior (ppm of N-meta < O-meta) is observed. However, concerning gas-phase data, such a discrepancy is reduced for both QM/FQ^b^ and QM/FDE/FQ^b^. Thus, it is probably related to the chosen combination of DFT functional and basis set rather than inaccuracies in the modeling of solvent effects.

Let us now comment on the results obtained for the coupling constants. Similarly to the previous case, the inclusion of the solvent moves the computed results toward the experiment for N-ortho, N-meta, O-meta, and O-ortho hydrogen atoms. However, in this case, for N-link and O-link atoms, the better agreement with the experiment is provided by the gas-phase calculations, while both QM/FQ^b^ and QM/FDE/FQ^b^ overestimate the coupling constants between these atoms. The large magnitudes computed for N-link and O-link coupling constants are consistent with the coupling constant of trans protons at a double bond. To explain such a result, we note that a zwitterion–quinone equilibrium is indeed possible for the studied molecule. Thus, our findings suggest that the zwitterionic form is stabilized by the external environment via intermolecular HB with the aqueous solution^94^ (vide infra for a more detailed analysis of the zwitterion–quinone equilibrium).

As a reference, the QM/FDE/FQFμ chemical shifts and spin-spin coupling constants of MOED in aqueous solution are reported in Figure 8. As already observed above, the agreement between the QM/FDE/FQ^b^ and QM/FDE/FQFμ spectra is almost perfect, with only a minor difference in the *J*_link_ value.

### 4.5 NMR Spectra in Nonaqueous Solutions

**Figure 9:**
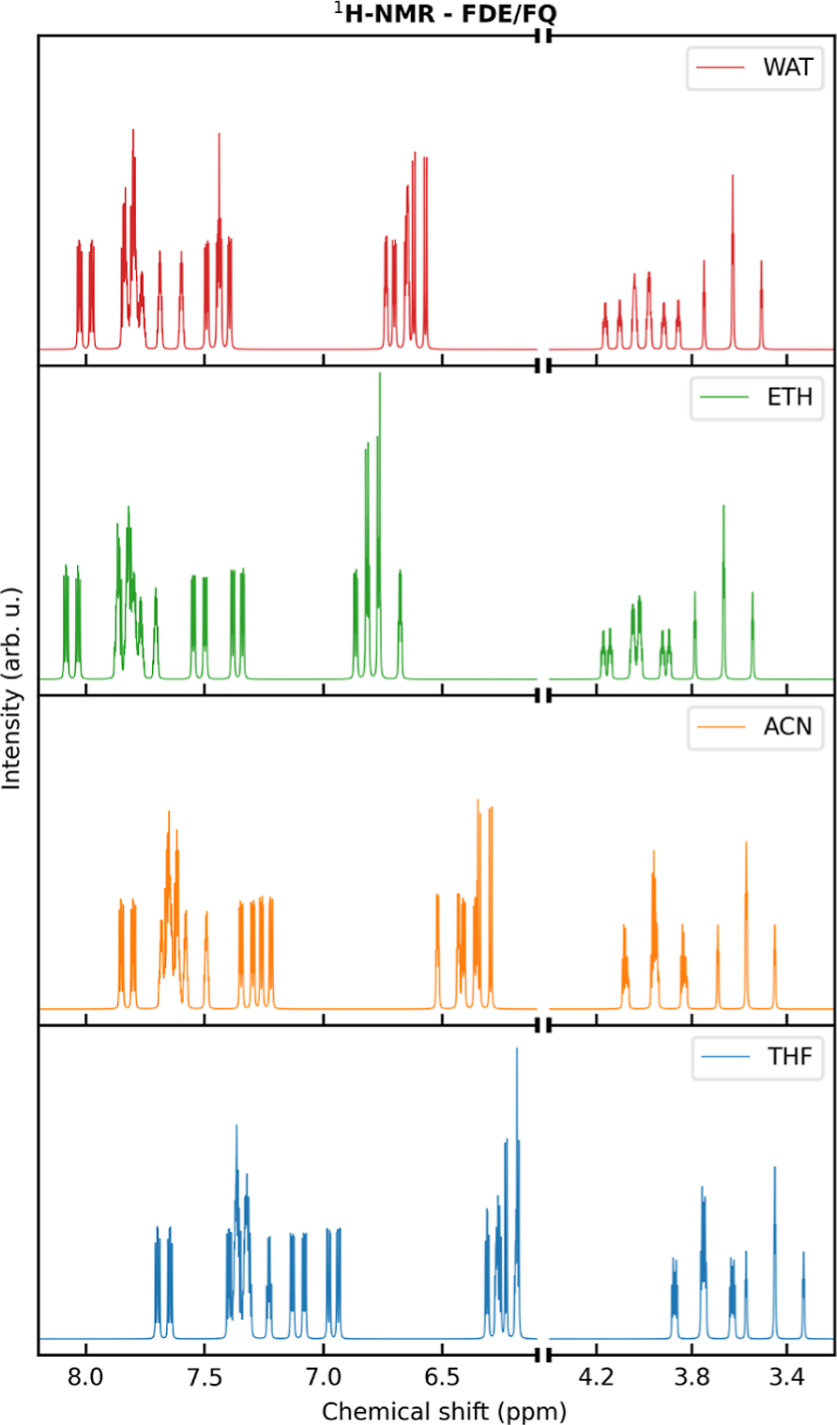
Computed QM/FDE/FQ^b 1^H NMR spectra of the MOED dissolved in selected solvents.

**Figure 10:**
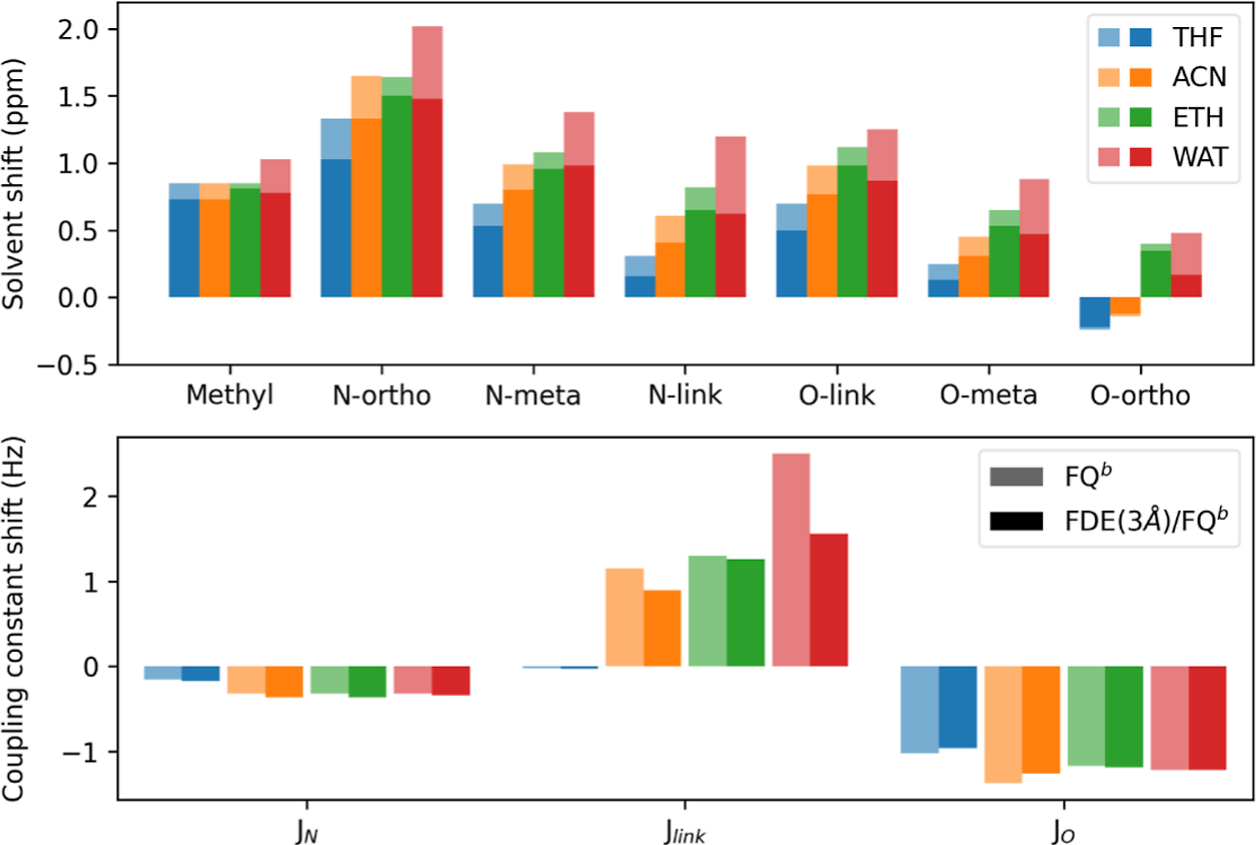
QM/FQ^b^ (light) and QM/FDE/FQ^b^ (dark) vacuo-to-solvent variations in chemical shift (ppm, upper panel) and *J* coupling constants (Hz, bottom panel).

As a final application of the novel methodology, we study ^1^H NMR spectra of MOED dissolved in nonaqueous solvents [ethanol (ETH), acetonitrile (ACN), and THF], of different polarities. We apply the same computational protocol outlined in the case of water, i.e. we resort to the MD simulations reported in ref (76) to sample the solute–solvent phase-space. For all solvents, both chemical shifts and spin–spin coupling constants are computed at the QM/FDE/FQ^b^ level, and the resulting ^1^H NMR spectra are reported in Figure 9. To analyze the effect of the intermediate FDE layer, in Figure 10, we report QM/FDE/FQ^b^ and QM/FQ^b^ solvent shifts for both chemical shifts and coupling constants as computed by taking the gas-phase value as a reference (raw data are given in Tables S16–S22 in the Supporting Information).

Figure 9 clearly shows that the inclusion of nonelectrostatic contributions via the intermediate FDE layer induces a nonuniform shielding of the protons with a consequent reduction of the chemical shifts. The only exception is given by the O-ortho chemical shifts for THF and ACN solutions, for which, however, the QM/FDE/FQ^b^ and QM/FQ^b^ difference is negligible. Such behavior, which has also been reported above for the case of aqueous solutions (see the previous section), can be justified by the density confinement effect associated with Pauli repulsion, which increases the electron density on the nuclei. Indeed, by comparing QM/FDE/FQ^b^ and QM/FQ^b^, the effect of nonelectrostatic interactions can be quantitatively estimated, while the difference between QM/FQ^b^ and gas-phase data can be correlated to the electrostatic and polarization contributions only. In particular, we note that on average, the inclusion of electrostatic effects yields an increase in the computed shieldings for all solvents. Such an effect increases by moving from THF (0.51 ppm) to ACN (0.76 ppm), to ETH (0.95 ppm), and to WAT (1.20 ppm), reflecting the different polarity associated with each solvent. For all solvents, the N-ortho proton reports the largest electrostatic shifts for which the largest depletion of the electron density is observed. This is also confirmed by the MOED dipole moment, which increases by moving from the gas phase (17.32 D) to the solution (THF: 28.85 D; ACN: 33.77 D; ETH: 37.66 D; WTR: 41.89 D). Let us now focus on the effect of nonelectrostatic interactions. From chemical intuition, we may speculate that they would dominate solute–solvent interactions for apolar solvents, such as THF. As stated above, we can evaluate their effect on the total computed shielding by subtracting QM/FDE/FQ^b^ and QM/FQ^b^ results. For all solvents and for all protons, the FDE shell yields a decrease in the proton shieldings, with the only exception reported for the O-ortho in THF and ACN. From a quantitative point of view, quantum confinement effects account for 30% for THF, 23% for ACN, and 14% for ETH, thus confirming our hypothesis. However, the largest contribution is provided by WAT (41%). This result highlights the necessity of including both electrostatic (polarization) and purely nonelectrostatic contributions in the case of highly polar and protic solvents.

In Figure 10, computed solvent effects on *J*-couplings are also reported. As can be noted, the results are much less sensitive to solvent polarity, and remarkably, the inclusion of nonelectrostatic contributions has an almost negligible effect, yielding on average a shift concerning QM/FQ^b^ data in the range between 0 and 5%. The largest solvent shift occurs for *J*_link_ in an aqueous solution (2.5 Hz for QM/FQ^b^, 1.6 Hz for QM/FDE/FQ^b^), for which the largest *J* variation by nonelectrostatic interactions (0.9 Hz) is also reported. The computed values of *J*_link_ both in the gas phase and in solution well-correlate with trans protons at a double bond.^97^ This indicates that the zwitterionic form is predominant in all situations. Notice also that such a character is accentuated by moving from the gas phase (16.62 Hz) to polar solvents such as water, as it is highlighted by the increase in the value of *J*_link_.^94^

These findings question our chemical intuition. In fact, one would expect the zwitterionic form to be more probable (stabilized) in polar solvents only, with the quinonoid form to be mainly present in gas phase and apolar solvents. Our results instead suggest that the zwitterionic–quinonoid equilibrium is almost unaffected by varying the polarity of the solvent because the spin–spin *J*_link_ coupling constants fall almost in the same range for all solvents. From an investigation of the ^13^C chemical shifts in Table S16 in the Supporting Information, it can be noticed that for the carbonyl carbon atom, a chemical shift of about 170–180 ppm is reported for all solvents. Such a value is compatible with a carbon atom doubly bonded to an oxygen atom, thus suggesting a quinonoid electronic form. These intriguing results, which might seem contradictory, have also been experimentally reported on similar structures,^94^ thus highlighting the complex electronic structure of MOED. To shed light on the zwitterion–quinone equilibrium in solution, we analyze MOED density through the quantum theory of atoms in molecules.^98,99^ In particular, we compute the delocalization indices (DI)^100,101^ between atom pairs A–B, which provide a quantitative measure of the electrons pairs that are shared among A and B. DI can thus be connected to the concept of bond order.^102−104^ In Table S15 in the Supporting Information, we report the DI numerical values for selected regions of MOED in various solvents (see also Section S2 in the Supporting Information). In summary, this analysis confirms the fact that the zwitterionic–quinonoid equilibrium is only partially affected by the nature of the solvent, with the zwitterionic form being stabilized in aqueous solution.

**Figure 11:**
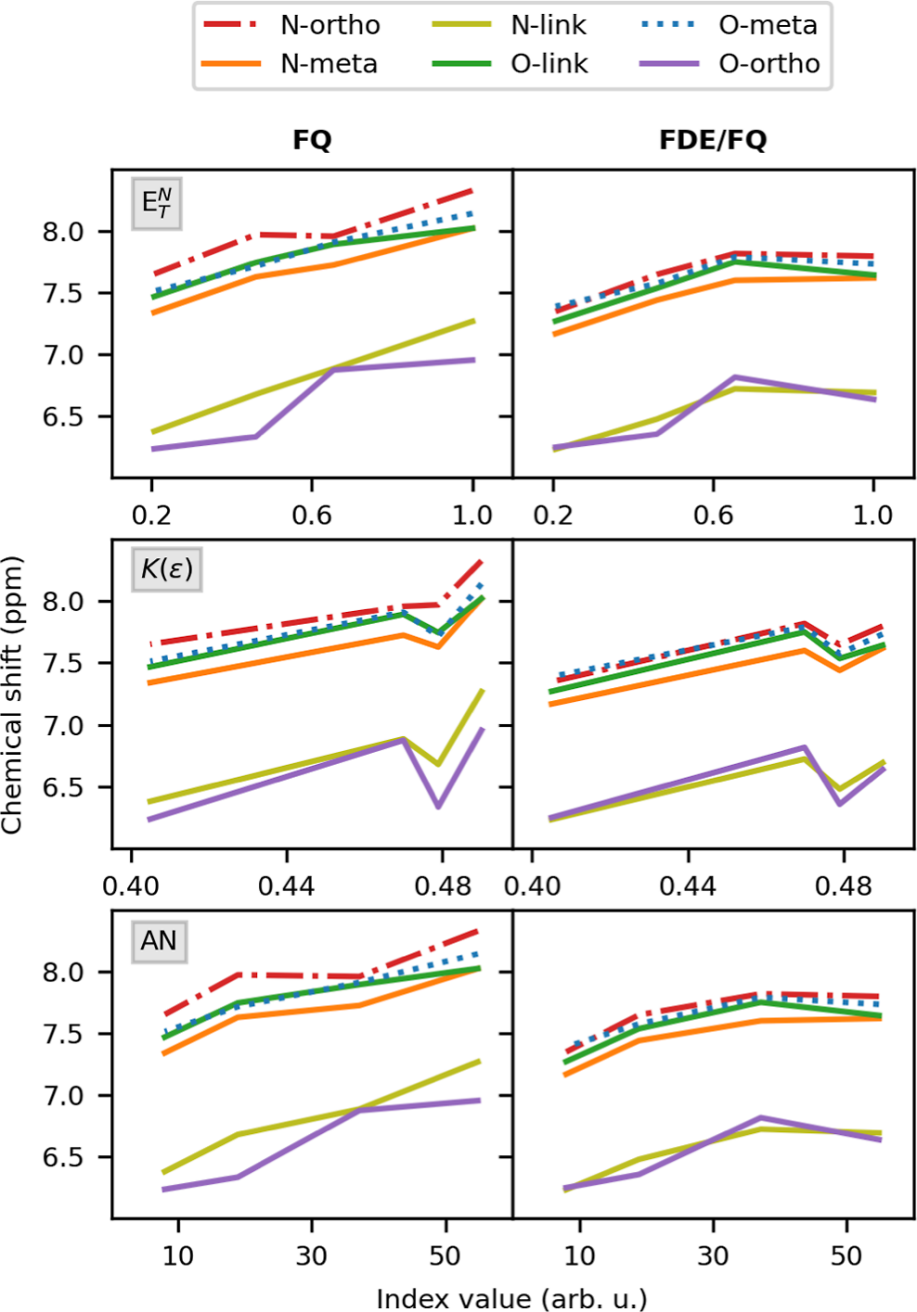
^1^H NMR chemical shifts computed at the QM/FQ^b^ and QM/FDE/FQ^b^ levels as a function of *E*_*T*_^*N*^, *K*(ε), and AN indexes for the selected solvents.

To further deepen our analysis of solvent effects on NMR signals, we attempt to correlate chemical shifts to solvents’ polarity.^105^ Three different solvent polarity scales are taken into account: *E*_*T*_^*N*^, Reichardt’s normalized parameters;^106,107^*K*(ε), Kirkwood–Bauer–Magat dielectric function;^108−110^ acceptor number (AN) by Mayer et al.^111^ The solvent’s polarities in the selected scales are given in Table 1, and their correlation plots with chemical shifts computed at the QM/FQ^b^ and QM/FDE/FQ^b^ levels of theory are depicted in Figure 11.

**Table 1:** Solvent Permittivity (ε), *E*_*T*_^*N*^, *K*(ε), and AN Indexes for the Studied Solvents (See Also the Text)

solvent	ε	*E*_*T*_^*N*^	*K*(ε)	AN
THF	7.4	0.207	0.405	8.0
ACN	35.7	0.460	0.479	18.9
ETH	24.8	0.654	0.470	37.1
WAT	78.3	1.000	0.490	54.8

For all solvents, a linear trend is observed for QM/FQ^b^*E*_*T*_^*N*^ and AN, with relevant deviations reported for O-ortho and N-ortho. For the *K*(ε) index, instead, such a linear trend deteriorates due to the interchange between ETH and ACN. In fact, *K*(ε) is based on the solvents’ static dielectric constant, and thus ACN (ε ∼ 35) is considered more polar than ETH (ε ∼ 24). By moving to QM/FDE/FQ^b^ calculations, any linear-like trend disappears for the *E*_*T*_^*N*^ and AN indices. This is due to the strong influence of nonelectrostatic contributions in the case of aqueous solutions, which results in a strong deviation from linearity. Such findings clearly indicate that solvent effects on NMR shieldings cannot be predicted by merely considering solvent dielectric properties due to the huge impact of nonelectrostatic interactions.

## 5 Summary and Conclusions

In this work, our recently developed QM/FDE/FQ method is extended to the simulation of NMR shielding and spin–spin coupling constants. The model performance is tested on MOED—Brooker’s merocyanine—dissolved in water by tuning different parameters: the size and the relaxation of the frozen density for the FDE intermediate layer and the inclusion of fluctuating dipoles in the classical environment so as to model anisotropic interactions. For the case of an aqueous solution, the inclusion of nonelectrostatic contributions appears to be critical for the correct reproduction of NMR spectra, as it is also demonstrated by the comparison with available experimental data.

The flexibility of QM/FDE/FQ is also highlighted by simulating NMR spectra of MOED in different solvents as an effective approach to simultaneously account for electrostatic, polarization, and Pauli repulsion effects. Our analysis, based on commonly exploited solvent polarity scales, shows that solvent effects cannot be reduced to the dielectric properties of the solvent only. This result highlights the necessity of a more sophisticated computational protocol to account for nonelectrostatic contributions, which may play a fundamental role in the simulation of magnetic properties.

According to the simulations presented in this paper, which are also confirmed by experimental measurements reported in the literature, the MOED shows a strong contribution from the zwitterionic form that is only slightly perturbed by solvent polarity. To further investigate this aspect, additional analysis of the topological properties of the electronic density in different solvents could be exploited. This might help in the rationalization of the trends in chemical shielding and spin–spin coupling constants reported for the different environments.

To conclude, QM/FDE/FQ and QM/FDE/FQFμ methods can be challenged to simulate NMR spectra of much more complicated systems, in particular as an investigation tool for the characterization of the NMR response of biological matrices,^112^ thanks to the synergy of the flexible atomistic description of the system coupled to the sophisticated, yet cost-effective, description of intermolecular interactions obtained through the quantum embedding. As a further development, we will investigate the extension of QM/FDE/FQ(Fμ) to the simulation of vibrational spectroscopy in the condensed phase.

## Supporting Information Available

Cartesian coordinates of MOED and technical details of the calculations; molecular structure of MOED with alternative labeling of the atoms; convergence analysis of selected hydrogen and carbon chemical shieldings and spin–spin coupling constants of MOED dissolved in various solvents; raw hydrogen chemical shifts and spin–spin coupling constants of MOED dissolved in aqueous solutions as computed at the QM/FQ^b^, QM/FDE(3 Å)/FQ^b^, and QM/FDE(5 Å)/FQ^b^ levels of theory; raw hydrogen chemical shifts and spin–spin coupling constants of MOED dissolved in aqueous solutions as computed at the QM/FDE(3 Å)/FQ^b^ level of theory with the adoption of freeze-and-thaw cycles; raw hydrogen chemical shifts and spin–spin coupling constants of MOED dissolved in aqueous solution as computed at the QM/MM and QM/FDE(3 Å)/MM levels of theory with different choices of the MM force field or of the FDE density, and of MOED in the gas phase; raw data of computed and experimental hydrogen chemical shifts and spin–spin coupling constants of MOED in the gas phase and dissolved in aqueous solution; bond order analysis of MOED in various solvents; and raw hydrogen and carbon chemical shifts and spin–spin coupling constants of MOED in the gas phase and dissolved in various solvents as computed at the QM/FQ^b^ and QM/FDE(3 Å)/FQ^b^ levels of theory (PDF)
