# Supplementary material for: Multiscale Frozen Density Embedding/Molecular Mechanics Approach for Simulating Magnetic Response Properties of Solvated Systems
Source: J Chem Theory Comput. 2023 Dec 18;20(1):266–79. doi: 10.1021/acs.jctc.3c00850 (PMC10782454; doi:10.1021/acs.jctc.3c00850)
Supplement: Supplementary file 1 — ct3c00850_si_001.pdf [file ct3c00850_si_001.pdf]

Supporting Information: Multiscale Frozen Density Embedding/Molecular Mechanics Approach for Simulating Magnetic Response Properties of Solvated Systems

Piero Lafiosca,[†] Federico Rossi,[†] Franco Egidi,[‡] Tommaso Giovannini,[†] and Chiara Cappelli[*,†]

†*Scuola Normale Superiore, Piazza dei Cavalieri 7, 56126 Pisa, Italy.*

‡*SCM*

E-mail: chiara.cappelli@sns.it

S1 Technical details

Table S1: MOED Cartesian coordinates in Angstrom optimized in gas phase.

C	-0.191645	0.322551	-0.632918
H	0.031355	-0.058449	-1.628918
C	0.225355	-0.391449	0.476082
H	-0.014645	0.022551	1.459082
C	0.935355	-1.608449	0.515082
C	1.271355	-2.183449	1.777082
C	1.352355	-2.322449	-0.648918
C	1.957355	-3.360449	1.886082
H	0.963355	-1.655449	2.684082
C	2.038355	-3.498449	-0.562918
H	1.118355	-1.920449	-1.634918
C	2.386355	-4.095449	0.715082
H	2.201355	-3.780449	2.861082
H	2.349355	-4.031449	-1.461918
O	3.018355	-5.179449	0.792082
C	-1.982645	3.399551	0.554082
H	-2.290645	3.925551	1.454082
C	-1.297645	2.219551	0.606082
H	-1.066645	1.816551	1.588082
C	-1.965645	3.376551	-1.785918
H	-2.260645	3.886551	-2.700918
C	-1.281645	2.197551	-1.798918
H	-1.023645	1.755551	-2.757918
C	-0.902645	1.544551	-0.585918
C	-3.046645	5.254551	-0.662918
H	-2.428645	6.021551	-1.138918
H	-3.978645	5.129551	-1.224918
H	-3.280645	5.561551	0.359082
N	-2.320645	3.983551	-0.622918

Keywords for the evaluation of FDE density:

```
basis
   core None
   type DZP
end
numericalquality Good
```

```
symmetry NOSYM
xc
   HYBRID B3LYP
end
Relativity Level=None
ExactDensity
```

Keywords for ground state calculation for QM/FDE/MM:

```
basis
   core None
   type DZP
end
numericalquality Good
symmetry NOSYM
xc
   HYBRID B3LYP
end
Relativity Level=None
ExactDensity
FDE
   PW91K
   GGAPOTXFD PBEx
   GGAPOTCFD PBEc
   RELAXCYCLES 3 # (freeze-and-thaw)
   FULLGRID
   ENERGY
end
Fragments
   frag1 t21.MERO
```

```
      frag2 t21.w type=FDE &
      FDEOPTIONS RELAX    # (freeze-and-thaw)
      XC GGA PBEx PBEc    # (freeze-and-thaw)
   subend
   end
   STOFit
```

Keywords for chemical shift calculations:

```
NMR
   OUT ISO
   U1K BEST
   ATOMS <list of atoms>
END
```

Keywords for spin-spin coupling constants calculations:

```
 NMRCoupling
  fc
  sd
  dso
  pso
  NUCLEI <list of nuclei>
 End
```

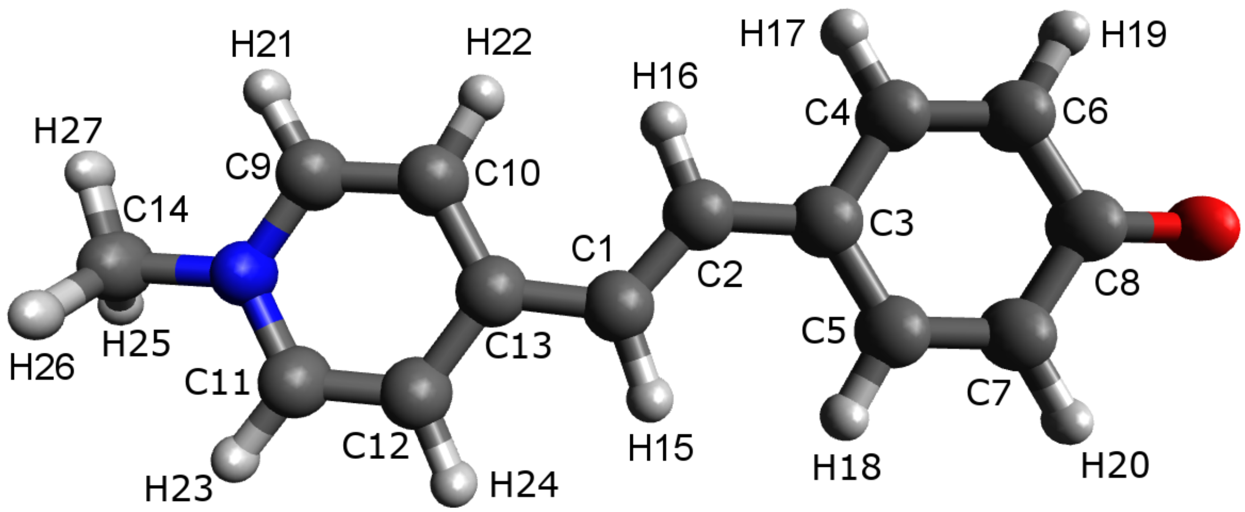

Figure S1: Brooker's merocyanine structure with the indication of atom types and labels

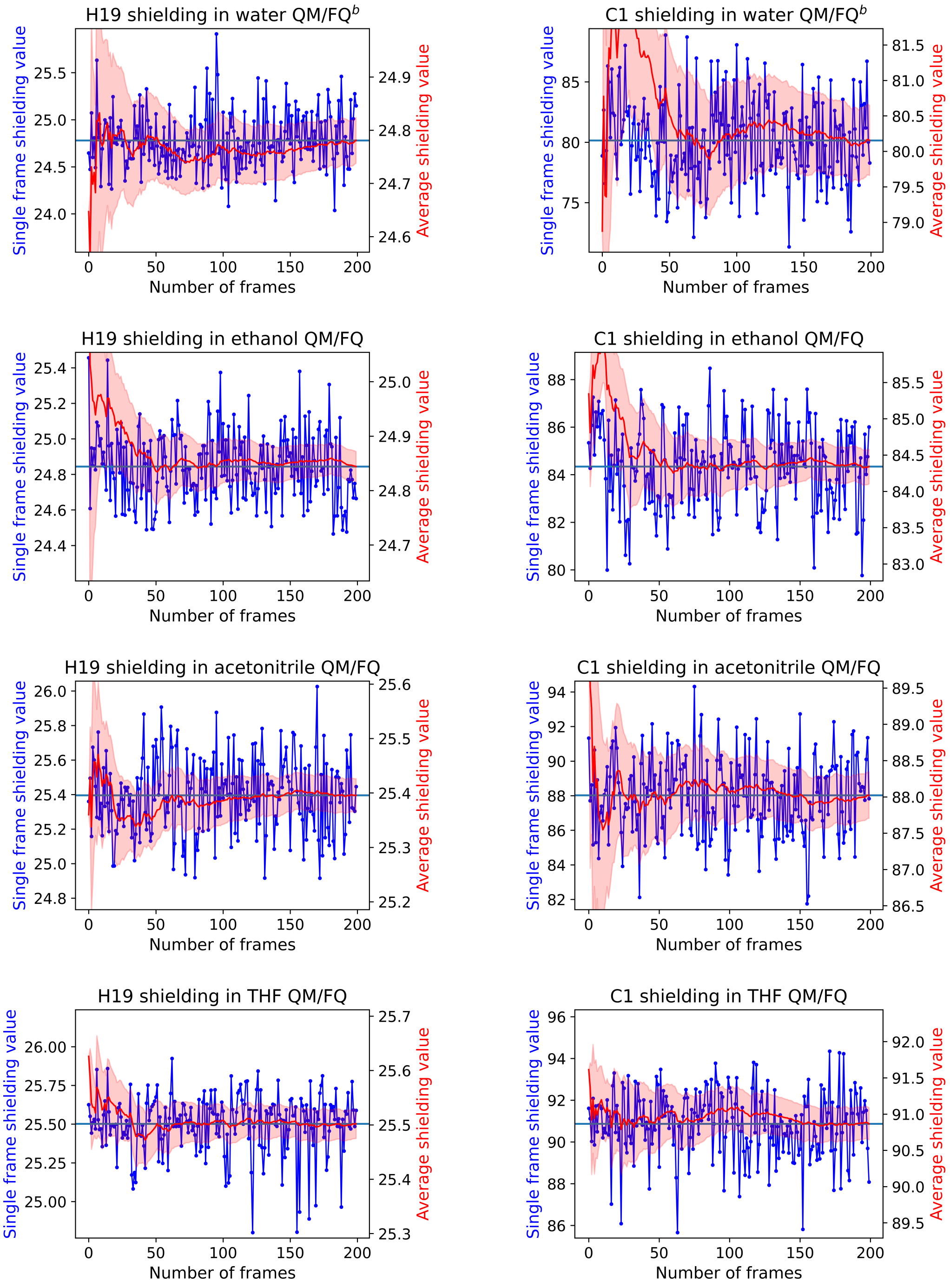

Figure S2: Convergence analysis over 200 snapshots for selected chemical shieldings of MOED in different solvents at the QM/FQ^b level of theory. The chemical shielding value of each snapshot is reported with blue dots, while the average for each number of snapshots is reported with a solid red line. The 95 % confidence interval for each number of snapshots is represented with the dark red area. The atoms labeling is reported in fig. S1.

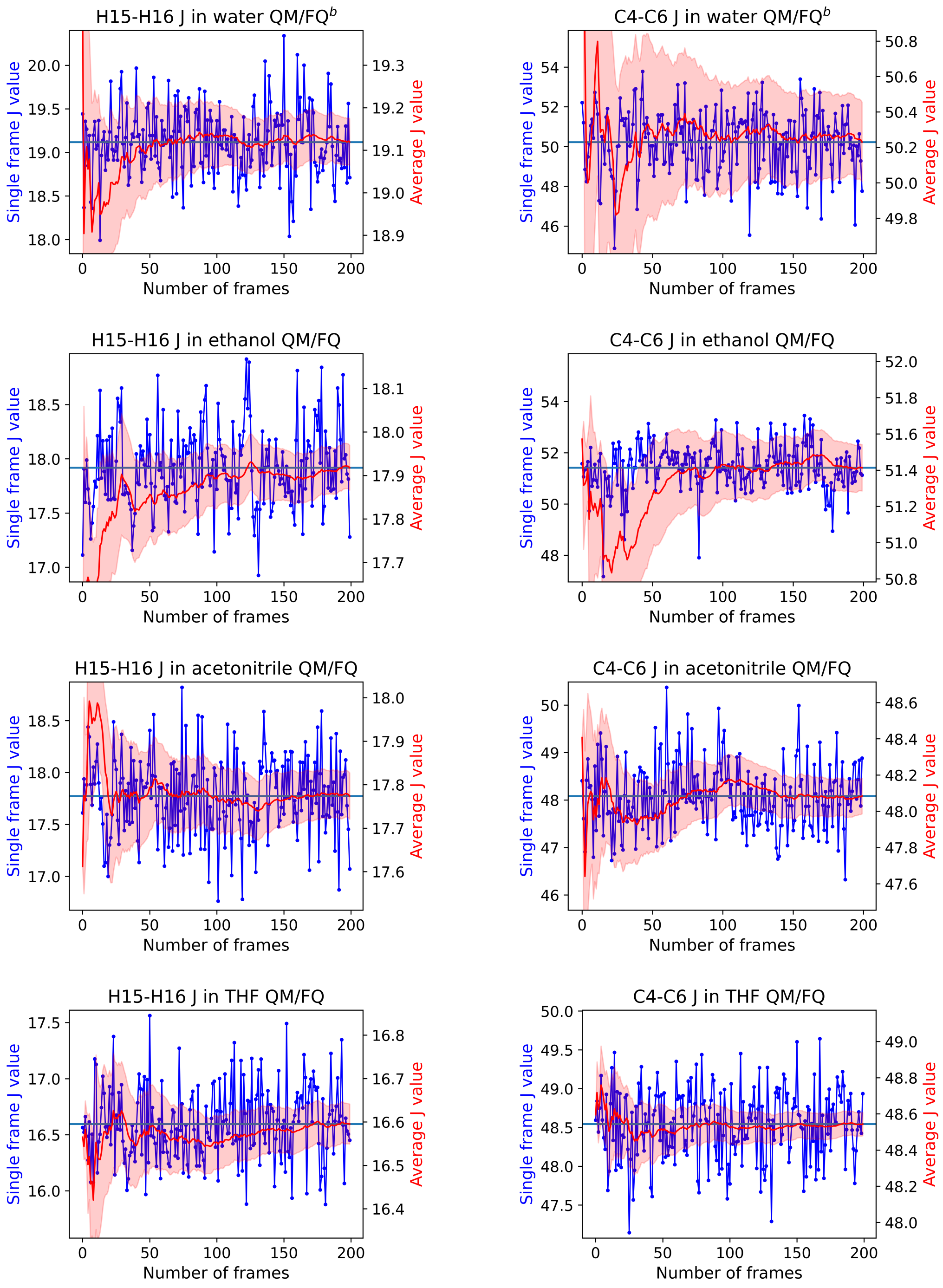

Figure S3: Convergence analysis over 200 snapshots for selected spin-spin coupling constants of MOED in different solvents at the QM/FQ^{b} level of theory. The spin-spin coupling constant of each snapshot is reported with blue dots, while the average for each number of snapshots is reported with a solid red line. The 95 % confidence interval for each number of snapshots is represented with the dark red area. The atoms labeling is reported in fig. S1.

Table S2: Computed H chemical shifts (ppm) at the QM/FQ[b], QM/FDE(3Å)/FQ[b] and QM/FDE(5Å)/FQ[b] levels for MOED dissolved in aqueous solution. The atoms labeling is reported in fig. S1.

Atom	FQ[b]	FDE(3Å)/FQ[b]	FDE(5Å)/FQ[b]
H(15)	7.267	6.692	6.598
H(16)	8.023	7.643	7.562
H(17)	7.848	7.466	7.408
H(18)	8.437	8.001	7.934
H(19)	6.919	6.596	6.622
H(20)	6.989	6.677	6.707
H(21)	8.301	7.784	7.596
H(22)	8.246	7.822	7.706
H(23)	8.356	7.811	7.622
H(24)	7.798	7.415	7.298
H(25)	4.246	4.041	3.944
H(26)	4.208	3.980	3.880
H(27)	3.927	3.628	3.522

Table S3: Computed H-H J couplings (Hz) at the QM/FQ[b] level for MOED dissolved in aqueous solution. The atoms labeling is reported in fig. S1.

Atom	H(15)	H(16)	H(17)	H(18)	H(19)	H(20)	H(21)	H(22)	H(23)	H(24)	H(25)	H(26)	H(27)
H(15)	0.00	19.12	0.51	0.16	-0.40	-0.09	0.69	-1.63	0.11	-0.91	0.44	0.38	-0.16
H(16)	19.12	0.00	-1.10	-1.59	0.16	0.71	-0.17	0.26	-0.35	0.48	-1.14	-1.02	-0.06
H(17)	0.51	-1.10	0.00	2.00	9.87	0.22	-0.02	0.46	-0.14	0.10	-0.35	-0.31	-0.02
H(18)	0.16	-1.59	2.00	0.00	0.35	10.09	-0.15	0.14	-0.05	0.41	-0.32	-0.28	-0.07
H(19)	-0.40	0.16	9.87	0.35	0.00	2.26	-0.04	-0.06	-0.09	-0.15	-0.01	-0.02	-0.05
H(20)	-0.09	0.71	0.22	10.09	2.26	0.00	-0.09	-0.15	-0.05	-0.03	-0.01	-0.02	-0.07
H(21)	0.69	-0.17	-0.02	-0.15	-0.04	-0.09	0.00	7.99	1.55	0.56	-1.86	-1.73	-0.80
H(22)	-1.63	0.26	0.46	0.14	-0.06	-0.15	7.99	0.00	0.72	1.93	0.64	0.57	-0.10
H(23)	0.11	-0.35	-0.14	-0.05	-0.09	-0.05	1.55	0.72	0.00	8.08	-1.71	-1.59	-0.63
H(24)	-0.91	0.48	0.10	0.41	-0.15	-0.03	0.56	1.93	8.08	0.00	0.48	0.39	0.02
H(25)	0.44	-1.14	-0.35	-0.32	-0.01	-0.01	-1.86	0.64	-1.71	0.48	0.00	-26.93	-24.46
H(26)	0.38	-1.02	-0.31	-0.28	-0.02	-0.02	-1.73	0.57	-1.59	0.39	-26.93	0.00	-24.29
H(27)	-0.16	-0.06	-0.02	-0.07	-0.05	-0.07	-0.80	-0.10	-0.63	0.02	-24.46	-24.29	0.00

Table S4: Computed H-H J couplings (Hz) at the QM/FDE(3Å)/FQ^b level for MOED dissolved in aqueous solution. The atoms labeling is reported in fig. S1.

Atom	H(15)	H(16)	H(17)	H(18)	H(19)	H(20)	H(21)	H(22)	H(23)	H(24)	H(25)	H(26)	H(27)
H(15)	0.00	18.18	0.16	-0.20	-0.21	0.12	0.68	-1.41	0.07	-0.67	0.06	0.04	-0.17
H(16)	18.18	0.00	-0.88	-1.41	0.14	0.78	-0.08	-0.02	-0.29	0.19	-0.71	-0.64	-0.06
H(17)	0.16	-0.88	0.00	2.22	9.83	-0.07	-0.00	0.37	-0.12	0.00	-0.20	-0.19	-0.02
H(18)	-0.20	-1.41	2.22	0.00	0.03	10.13	-0.13	0.04	-0.04	0.32	-0.18	-0.17	-0.07
H(19)	-0.21	0.14	9.83	0.03	0.00	2.06	-0.03	0.03	-0.05	-0.09	-0.16	-0.15	-0.06
H(20)	0.12	0.78	-0.07	10.13	2.06	0.00	-0.08	-0.07	-0.02	0.03	-0.16	-0.14	-0.07
H(21)	0.68	-0.08	-0.00	-0.13	-0.03	-0.08	0.00	7.97	1.63	0.33	-1.51	-1.42	-0.77
H(22)	-1.41	-0.02	0.37	0.04	0.03	-0.07	7.97	0.00	0.44	2.03	0.39	0.35	-0.12
H(23)	0.07	-0.29	-0.12	-0.04	-0.05	-0.02	1.63	0.44	0.00	8.07	-1.30	-1.23	-0.57
H(24)	-0.67	0.19	0.00	0.32	-0.09	0.03	0.33	2.03	8.07	0.00	0.23	0.17	-0.01
H(25)	0.06	-0.71	-0.20	-0.18	-0.16	-0.16	-1.51	0.39	-1.30	0.23	0.00	-25.18	-24.13
H(26)	0.04	-0.64	-0.19	-0.17	-0.15	-0.14	-1.42	0.35	-1.23	0.17	-25.18	0.00	-24.04
H(27)	-0.17	-0.06	-0.02	-0.07	-0.06	-0.07	-0.77	-0.12	-0.57	-0.01	-24.13	-24.04	0.00

Table S5: Computed H-H J couplings (Hz) at the QM/FDE(5Å)/FQ^b level for MOED dissolved in aqueous solution. The atoms labeling is reported in fig. S1.

Atom	H(15)	H(16)	H(17)	H(18)	H(19)	H(20)	H(21)	H(22)	H(23)	H(24)	H(25)	H(26)	H(27)
H(15)	0.00	17.76	0.08	-0.28	-0.20	0.13	0.71	-1.34	0.09	-0.61	-0.08	-0.09	-0.18
H(16)	17.76	0.00	-0.84	-1.38	0.20	0.86	-0.07	-0.11	-0.29	0.10	-0.55	-0.50	-0.05
H(17)	0.08	-0.84	0.00	2.28	9.88	-0.14	0.00	0.34	-0.12	-0.02	-0.16	-0.15	-0.02
H(18)	-0.28	-1.38	2.28	0.00	-0.05	10.21	-0.12	0.01	-0.03	0.29	-0.15	-0.13	-0.07
H(19)	-0.20	0.20	9.88	-0.05	0.00	2.01	-0.01	0.05	-0.02	-0.08	-0.21	-0.20	-0.06
H(20)	0.13	0.86	-0.14	10.21	2.01	0.00	-0.07	-0.05	0.01	0.04	-0.20	-0.18	-0.07
H(21)	0.71	-0.07	0.00	-0.12	-0.01	-0.07	0.00	7.95	1.64	0.23	-1.36	-1.29	-0.77
H(22)	-1.34	-0.11	0.34	0.01	0.05	-0.05	7.95	0.00	0.32	2.06	0.29	0.26	-0.12
H(23)	0.09	-0.29	-0.12	-0.03	-0.02	0.01	1.64	0.32	0.00	8.06	-1.14	-1.09	-0.55
H(24)	-0.61	0.10	-0.02	0.29	-0.08	0.04	0.23	2.06	8.06	0.00	0.13	0.08	-0.02
H(25)	-0.08	-0.55	-0.16	-0.15	-0.21	-0.20	-1.36	0.29	-1.14	0.13	0.00	-24.52	-23.77
H(26)	-0.09	-0.50	-0.15	-0.13	-0.20	-0.18	-1.29	0.26	-1.09	0.08	-24.52	0.00	-23.70
H(27)	-0.18	-0.05	-0.02	-0.07	-0.06	-0.07	-0.77	-0.12	-0.55	-0.02	-23.77	-23.70	0.00

Table S6: Computed H-H chemical shifts (ppm) and J couplings (Hz) at the QM/FDE(3Å)/FQ^b level for MOED dissolved in aqueous solution with the adoption of freeze-and-thaw cycles. The atoms labeling is reported in fig. S1.

Atom	Shift	H(15)	H(16)	H(17)	H(18)	H(19)	H(20)	H(21)	H(22)	H(23)	H(24)	H(25)	H(26)	H(27)
H(15)	6.701	0.00	18.20	0.17	-0.19	-0.22	0.11	0.68	-1.41	0.07	-0.67	0.07	0.05	-0.17
H(16)	7.65	18.20	0.00	-0.87	-1.41	0.14	0.78	-0.08	-0.01	-0.29	0.20	-0.71	-0.64	-0.06
H(17)	7.475	0.17	-0.87	0.00	2.22	9.83	-0.07	-0.00	0.37	-0.12	0.01	-0.21	-0.19	-0.02
H(18)	8.008	-0.19	-1.41	2.22	0.00	0.03	10.13	-0.13	0.05	-0.04	0.32	-0.19	-0.17	-0.07
H(19)	6.608	-0.22	0.14	9.83	0.03	0.00	2.07	-0.03	0.03	-0.05	-0.09	-0.16	-0.15	-0.06
H(20)	6.688	0.11	0.78	-0.07	10.13	2.07	0.00	-0.09	-0.07	-0.02	0.03	-0.15	-0.14	-0.07
H(21)	7.789	0.68	-0.08	-0.00	-0.13	-0.03	-0.09	0.00	7.97	1.63	0.33	-1.51	-1.42	-0.77
H(22)	7.829	-1.41	-0.01	0.37	0.05	0.03	-0.07	7.97	0.00	0.44	2.03	0.39	0.35	-0.12
H(23)	7.817	0.07	-0.29	-0.12	-0.04	-0.05	-0.02	1.63	0.44	0.00	8.08	-1.30	-1.23	-0.57
H(24)	7.42	-0.67	0.20	0.01	0.32	-0.09	0.03	0.33	2.03	8.08	0.00	0.23	0.17	-0.01
H(25)	4.043	0.07	-0.71	-0.21	-0.19	-0.16	-0.15	-1.51	0.39	-1.30	0.23	0.00	-25.18	-24.13
H(26)	3.982	0.05	-0.64	-0.19	-0.17	-0.15	-0.14	-1.42	0.35	-1.23	0.17	-25.18	0.00	-24.04
H(27)	3.63	-0.17	-0.06	-0.02	-0.07	-0.06	-0.07	-0.77	-0.12	-0.57	-0.01	-24.13	-24.04	0.00

Table S7: Chemical shifts (ppm) of protons in water using different MM models. The value for each nucleus is accompanied by the difference with respect to the gas-phase calculation obtained as $\Delta = \delta_i - \delta_{vac}$ where δ_i and δ_{vac} are the chemical shift calculated with the indicated model and in gas-phase, respectively. Besides that, also the chemical shifts after the inclusion of the FDE shell (3Å) are presented, together with the difference with respect to the values of the QM/MM calculation as $\Delta\Delta = \delta_{i,FDE/MM} - \delta_{i,MM}$. The atoms labeling is reported in fig. S1.

Atom	Vac	TIP3P		FQ^a		FQ^b		FQFμ	
		Δ TIP3P	ΔΔ FDE/TIP3P	Δ FQ^a	ΔΔ FDE/FQ^a	Δ FQ^b	ΔΔ FDE/FQ^b	Δ FQFμ	ΔΔ FDE/FQFμ
H(15)	6.071	6.734 (0.663)	6.566 (-0.167)	6.625 (0.554)	6.525 (-0.099)	7.267 (1.196)	6.692 (-0.575)	7.378 (1.307)	6.752 (-0.626)
H(16)	6.772	7.695 (0.923)	7.532 (-0.163)	7.586 (0.814)	7.481 (-0.106)	8.023 (1.251)	7.643 (-0.380)	8.042 (1.270)	7.681 (-0.361)
H(17)	7.018	7.497 (0.479)	7.386 (-0.111)	7.432 (0.414)	7.353 (-0.079)	7.848 (0.830)	7.466 (-0.381)	7.896 (0.878)	7.501 (-0.395)
H(18)	7.497	8.058 (0.561)	7.910 (-0.149)	7.983 (0.486)	7.874 (-0.109)	8.437 (0.940)	8.001 (-0.436)	8.485 (0.988)	8.044 (-0.441)
H(19)	6.442	6.674 (0.232)	6.628 (-0.046)	6.675 (0.233)	6.647 (-0.029)	6.919 (0.477)	6.596 (-0.323)	6.890 (0.448)	6.577 (-0.313)
H(20)	6.507	6.750 (0.243)	6.714 (-0.035)	6.750 (0.243)	6.731 (-0.018)	6.989 (0.482)	6.677 (-0.312)	6.968 (0.461)	6.663 (-0.305)
H(21)	6.347	7.719 (1.372)	7.543 (-0.176)	7.505 (1.158)	7.455 (-0.051)	8.301 (1.954)	7.784 (-0.517)	8.468 (2.121)	7.890 (-0.578)
H(22)	6.789	7.829 (1.040)	7.670 (-0.160)	7.689 (0.900)	7.607 (-0.082)	8.246 (1.457)	7.822 (-0.425)	8.311 (1.522)	7.881 (-0.430)
H(23)	6.287	7.749 (1.462)	7.559 (-0.190)	7.521 (1.234)	7.468 (-0.053)	8.356 (2.069)	7.811 (-0.545)	8.523 (2.236)	7.922 (-0.602)
H(24)	6.489	7.405 (0.916)	7.260 (-0.145)	7.276 (0.787)	7.208 (-0.068)	7.798 (1.309)	7.415 (-0.383)	7.879 (1.390)	7.478 (-0.401)
H(25)	3.211	3.948 (0.737)	3.908 (-0.040)	3.842 (0.631)	3.862 (0.019)	4.246 (1.035)	4.041 (-0.206)	4.325 (1.114)	4.099 (-0.226)
H(26)	3.212	3.902 (0.690)	3.849 (-0.053)	3.798 (0.586)	3.804 (0.006)	4.208 (0.996)	3.980 (-0.228)	4.282 (1.070)	4.038 (-0.244)
H(27)	2.877	3.593 (0.716)	3.489 (-0.104)	3.483 (0.606)	3.439 (-0.044)	3.927 (1.050)	3.628 (-0.299)	4.015 (1.138)	3.693 (-0.322)

Table S8: Computed H-H J couplings (Hz) at the QM level for MOED in gas-phase. The atoms labeling is reported in fig. S1.

Atom	H(15)	H(16)	H(17)	H(18)	H(19)	H(20)	H(21)	H(22)	H(23)	H(24)	H(25)	H(26)	H(27)
H(15)	0.00	16.62	0.00	0.00	-1.05	-0.69	1.48	-1.28	0.96	-0.82	-1.22	-1.12	0.00
H(16)	16.62	0.00	-1.91	-2.17	1.93	2.80	-0.72	-0.72	-1.28	0.00	0.92	0.83	0.00
H(17)	0.00	-1.91	0.00	1.98	11.00	0.00	0.00	0.00	-0.53	0.00	0.00	0.00	0.00
H(18)	0.00	-2.17	1.98	0.00	0.00	11.37	0.00	0.00	0.00	0.00	0.00	0.00	0.00
H(19)	-1.05	1.93	11.00	0.00	0.00	0.61	0.51	0.00	0.93	0.00	-1.06	-0.98	0.00
H(20)	-0.69	2.80	0.00	11.37	0.61	0.00	0.00	0.00	0.78	0.00	-0.98	-0.90	0.00
H(21)	1.48	-0.72	0.00	0.00	0.51	0.00	0.00	8.25	1.43	0.00	0.00	0.00	-0.96
H(22)	-1.28	-0.72	0.00	0.00	0.00	0.00	8.25	0.00	0.00	2.30	0.00	0.00	0.00
H(23)	0.96	-1.28	-0.53	0.00	0.93	0.78	1.43	0.00	0.00	8.46	0.00	0.00	0.00
H(24)	-0.82	0.00	0.00	0.00	0.00	0.00	0.00	2.30	8.46	0.00	0.00	0.00	0.00
H(25)	-1.22	0.92	0.00	0.00	-1.06	-0.98	0.00	0.00	0.00	0.00	0.00	-23.17	-23.67
H(26)	-1.12	0.83	0.00	0.00	-0.98	-0.90	0.00	0.00	0.00	0.00	-23.17	0.00	-23.78
H(27)	0.00	0.00	0.00	0.00	0.00	0.00	-0.96	0.00	0.00	0.00	-23.67	-23.78	0.00

Table S9: Computed H-H J couplings (Hz) at the QM/TIP3P (upper panel) and QM/FDE(3Å)/TIP3P (bottom panel) levels for MOED in aqueous solution. The atoms labeling is reported in fig. S1.

						QM/TIP3P							
Atom	H(15)	H(16)	H(17)	H(18)	H(19)	H(20)	H(21)	H(22)	H(23)	H(24)	H(25)	H(26)	H(27)
H(15)	0.00	17.73	0.10	-0.32	-0.21	0.12	0.69	-1.37	0.07	-0.72	-0.02	-0.03	-0.18
H(16)	17.73	0.00	-0.94	-1.39	0.16	0.81	-0.07	-0.13	-0.28	0.13	-0.63	-0.57	-0.05
H(17)	0.10	-0.94	0.00	2.22	9.88	-0.12	0.00	0.36	-0.12	-0.01	-0.19	-0.17	-0.02
H(18)	-0.32	-1.39	2.22	0.00	-0.02	10.21	-0.12	0.03	-0.03	0.30	-0.17	-0.15	-0.07
H(19)	-0.21	0.16	9.88	-0.02	0.00	2.08	-0.02	0.04	-0.04	-0.09	-0.18	-0.17	-0.06
H(20)	0.12	0.81	-0.12	10.21	2.08	0.00	-0.08	-0.06	-0.01	0.04	-0.17	-0.16	-0.07
H(21)	0.69	-0.07	0.00	-0.12	-0.02	-0.08	0.00	7.98	1.64	0.27	-1.44	-1.36	-0.85
H(22)	-1.37	-0.13	0.36	0.03	0.04	-0.06	7.98	0.00	0.36	2.04	0.33	0.30	-0.12
H(23)	0.07	-0.28	-0.12	-0.03	-0.04	-0.01	1.64	0.36	0.00	8.08	-1.26	-1.21	-0.58
H(24)	-0.72	0.13	-0.01	0.30	-0.09	0.04	0.27	2.04	8.08	0.00	0.17	0.12	-0.02
H(25)	-0.02	-0.63	-0.19	-0.17	-0.18	-0.17	-1.44	0.33	-1.26	0.17	0.00	-25.41	-24.24
H(26)	-0.03	-0.57	-0.17	-0.15	-0.17	-0.16	-1.36	0.30	-1.21	0.12	-25.41	0.00	-24.15
H(27)	-0.18	-0.05	-0.02	-0.07	-0.06	-0.07	-0.85	-0.12	-0.58	-0.02	-24.24	-24.15	0.00
						QM/FDE(3Å)/TIP3P							
H(15)	0.00	17.78	0.05	-0.31	-0.20	0.13	0.72	-1.33	0.09	-0.61	-0.12	-0.12	-0.18
H(16)	17.78	0.00	-0.86	-1.39	0.22	0.90	-0.07	-0.15	-0.30	0.09	-0.51	-0.47	-0.05
H(17)	0.05	-0.86	0.00	2.31	9.96	-0.16	0.01	0.33	-0.12	-0.03	-0.15	-0.14	-0.02
H(18)	-0.31	-1.39	2.31	0.00	-0.07	10.31	-0.12	0.01	-0.03	0.28	-0.14	-0.13	-0.07
H(19)	-0.20	0.22	9.96	-0.07	0.00	2.00	-0.01	0.05	-0.01	-0.08	-0.23	-0.21	-0.06
H(20)	0.13	0.90	-0.16	10.31	2.00	0.00	-0.06	-0.05	0.01	0.04	-0.21	-0.19	-0.07
H(21)	0.72	-0.07	0.01	-0.12	-0.01	-0.06	0.00	7.99	1.66	0.21	-1.34	-1.26	-0.79
H(22)	-1.33	-0.15	0.33	0.01	0.05	-0.05	7.99	0.00	0.29	2.08	0.27	0.25	-0.13
H(23)	0.09	-0.30	-0.12	-0.03	-0.01	0.01	1.66	0.29	0.00	8.10	-1.11	-1.07	-0.56
H(24)	-0.61	0.09	-0.03	0.28	-0.08	0.04	0.21	2.08	8.10	0.00	0.11	0.06	-0.03
H(25)	-0.12	-0.51	-0.15	-0.14	-0.23	-0.21	-1.34	0.27	-1.11	0.11	0.00	-24.53	-23.90
H(26)	-0.12	-0.47	-0.14	-0.13	-0.21	-0.19	-1.26	0.25	-1.07	0.06	-24.53	0.00	-23.86
H(27)	-0.18	-0.05	-0.02	-0.07	-0.06	-0.07	-0.79	-0.13	-0.56	-0.03	-23.90	-23.86	0.00

Table S10: Computed H-H *J* couplings (Hz) at the QM/FQ[a] (upper panel) and QM/FDE(3Å)/FQ[a] (bottom panel) levels for MOED in aqueous solution. The atoms labeling is reported in fig. S1.

QM/FQ[a]													
Atom	H(15)	H(16)	H(17)	H(18)	H(19)	H(20)	H(21)	H(22)	H(23)	H(24)	H(25)	H(26)	H(27)
H(15)	0.00	17.37	0.03	-0.40	-0.21	0.12	0.73	-1.31	0.10	-0.68	-0.17	-0.16	-0.18
H(16)	17.37	0.00	-0.95	-1.38	0.23	0.90	-0.07	-0.24	-0.30	0.05	-0.48	-0.44	-0.05
H(17)	0.03	-0.95	0.00	2.26	9.96	-0.18	0.01	0.33	-0.12	-0.04	-0.15	-0.14	-0.02
H(18)	-0.40	-1.38	2.26	0.00	-0.09	10.31	-0.12	0.00	-0.03	0.28	-0.14	-0.12	-0.07
H(19)	-0.21	0.23	9.96	-0.09	0.00	2.01	-0.00	0.05	0.00	-0.09	-0.24	-0.22	-0.06
H(20)	0.12	0.90	-0.18	10.31	2.01	0.00	-0.06	-0.05	0.02	0.03	-0.22	-0.20	-0.07
H(21)	0.73	-0.07	0.01	-0.12	-0.00	-0.06	0.00	8.00	1.66	0.16	-1.29	-1.23	-0.88
H(22)	-1.31	-0.24	0.33	0.00	0.05	-0.05	8.00	0.00	0.24	2.08	0.24	0.22	-0.13
H(23)	0.10	-0.30	-0.12	-0.03	0.00	0.02	1.66	0.24	0.00	8.10	-1.11	-1.08	-0.56
H(24)	-0.68	0.05	-0.04	0.28	-0.09	0.03	0.16	2.08	8.10	0.00	0.08	0.03	-0.03
H(25)	-0.17	-0.48	-0.15	-0.14	-0.24	-0.22	-1.29	0.24	-1.11	0.08	0.00	-24.95	-24.13
H(26)	-0.16	-0.44	-0.14	-0.12	-0.22	-0.20	-1.23	0.22	-1.08	0.03	-24.95	0.00	-24.05
H(27)	-0.18	-0.05	-0.02	-0.07	-0.06	-0.07	-0.88	-0.13	-0.56	-0.03	-24.13	-24.05	0.00
QM/FDE(3Å)/FQ[a]													
H(15)	0.00	17.64	0.02	-0.34	-0.20	0.13	0.74	-1.31	0.11	-0.59	-0.18	-0.17	-0.18
H(16)	17.64	0.00	-0.86	-1.39	0.26	0.95	-0.08	-0.19	-0.31	0.05	-0.44	-0.40	-0.05
H(17)	0.02	-0.86	0.00	2.33	10.03	-0.19	0.01	0.32	-0.12	-0.04	-0.13	-0.13	-0.02
H(18)	-0.34	-1.39	2.33	0.00	-0.09	10.39	-0.12	-0.00	-0.03	0.28	-0.12	-0.11	-0.07
H(19)	-0.20	0.26	10.03	-0.09	0.00	1.95	0.00	0.06	0.01	-0.08	-0.26	-0.24	-0.06
H(20)	0.13	0.95	-0.19	10.39	1.95	0.00	-0.05	-0.05	0.03	0.03	-0.24	-0.22	-0.07
H(21)	0.74	-0.08	0.01	-0.12	0.00	-0.05	0.00	8.00	1.66	0.17	-1.27	-1.21	-0.80
H(22)	-1.31	-0.19	0.32	-0.00	0.06	-0.05	8.00	0.00	0.24	2.10	0.23	0.21	-0.13
H(23)	0.11	-0.31	-0.12	-0.03	0.01	0.03	1.66	0.24	0.00	8.11	-1.04	-1.01	-0.55
H(24)	-0.59	0.05	-0.04	0.28	-0.08	0.03	0.17	2.10	8.11	0.00	0.07	0.02	-0.03
H(25)	-0.18	-0.44	-0.13	-0.12	-0.26	-0.24	-1.27	0.23	-1.04	0.07	0.00	-24.35	-23.84
H(26)	-0.17	-0.40	-0.13	-0.11	-0.24	-0.22	-1.21	0.21	-1.01	0.02	-24.35	0.00	-23.81
H(27)	-0.18	-0.05	-0.02	-0.07	-0.06	-0.07	-0.80	-0.13	-0.55	-0.03	-23.84	-23.81	0.00

Table S11: Computed H-H J couplings (Hz) at the QM/FQFμ (upper panel) and QM/FDE(3Å)/FQFμ (bottom panel) levels for MOED in aqueous solution. The atoms labeling is reported in fig. S1.

						QM/FQFμ							
Atom	H(15)	H(16)	H(17)	H(18)	H(19)	H(20)	H(21)	H(22)	H(23)	H(24)	H(25)	H(26)	H(27)
H(15)	0.00	19.45	0.58	0.25	-0.43	-0.11	0.70	-1.69	0.15	-0.95	0.55	0.47	-0.16
H(16)	19.45	0.00	-1.12	-1.62	0.17	0.71	-0.21	0.35	-0.39	0.56	-1.24	-1.11	-0.06
H(17)	0.58	-1.12	0.00	1.98	9.85	0.26	-0.03	0.48	-0.15	0.11	-0.37	-0.33	-0.02
H(18)	0.25	-1.62	1.98	0.00	0.38	10.04	-0.16	0.16	-0.07	0.42	-0.34	-0.30	-0.07
H(19)	-0.43	0.17	9.85	0.38	0.00	2.31	-0.04	-0.07	-0.09	-0.16	0.01	-0.00	-0.05
H(20)	-0.11	0.71	0.26	10.04	2.31	0.00	-0.09	-0.15	-0.05	-0.03	-0.00	-0.01	-0.07
H(21)	0.70	-0.21	-0.03	-0.16	-0.04	-0.09	0.00	8.02	1.51	0.64	-1.98	-1.84	-0.77
H(22)	-1.69	0.35	0.48	0.16	-0.07	-0.15	8.02	0.00	0.82	1.92	0.74	0.65	-0.10
H(23)	0.15	-0.39	-0.15	-0.07	-0.09	-0.05	1.51	0.82	0.00	8.11	-1.83	-1.70	-0.65
H(24)	-0.95	0.56	0.11	0.42	-0.16	-0.03	0.64	1.92	8.11	0.00	0.57	0.48	0.03
H(25)	0.55	-1.24	-0.37	-0.34	0.01	-0.00	-1.98	0.74	-1.83	0.57	0.00	-27.45	-24.48
H(26)	0.47	-1.11	-0.33	-0.30	-0.00	-0.01	-1.84	0.65	-1.70	0.48	-27.45	0.00	-24.29
H(27)	-0.16	-0.06	-0.02	-0.07	-0.05	-0.07	-0.77	-0.10	-0.65	0.03	-24.48	-24.29	0.00
						QM/FDE(3Å)/FQFμ							
H(15)	0.00	18.38	0.22	-0.14	-0.23	0.10	0.68	-1.45	0.07	-0.71	0.14	0.11	-0.17
H(16)	18.38	0.00	-0.90	-1.43	0.13	0.75	-0.09	0.03	-0.29	0.25	-0.79	-0.71	-0.06
H(17)	0.22	-0.90	0.00	2.19	9.77	-0.04	-0.00	0.38	-0.12	0.02	-0.23	-0.21	-0.02
H(18)	-0.14	-1.43	2.19	0.00	0.07	10.06	-0.13	0.06	-0.04	0.33	-0.21	-0.18	-0.07
H(19)	-0.23	0.13	9.77	0.07	0.00	2.07	-0.04	0.02	-0.06	-0.10	-0.13	-0.13	-0.05
H(20)	0.10	0.75	-0.04	10.06	2.07	0.00	-0.09	-0.08	-0.03	0.03	-0.13	-0.12	-0.07
H(21)	0.68	-0.09	-0.00	-0.13	-0.04	-0.09	0.00	7.96	1.61	0.38	-1.59	-1.49	-0.76
H(22)	-1.45	0.03	0.38	0.06	0.02	-0.08	7.96	0.00	0.50	2.01	0.44	0.40	-0.11
H(23)	0.07	-0.29	-0.12	-0.04	-0.06	-0.03	1.61	0.50	0.00	8.06	-1.39	-1.30	-0.58
H(24)	-0.71	0.25	0.02	0.33	-0.10	0.03	0.38	2.01	8.06	0.00	0.29	0.22	-0.00
H(25)	0.14	-0.79	-0.23	-0.21	-0.13	-0.13	-1.59	0.44	-1.39	0.29	0.00	-25.45	-24.18
H(26)	0.11	-0.71	-0.21	-0.18	-0.13	-0.12	-1.49	0.40	-1.30	0.22	-25.45	0.00	-24.07
H(27)	-0.17	-0.06	-0.02	-0.07	-0.05	-0.07	-0.76	-0.11	-0.58	-0.00	-24.18	-24.07	0.00

Table S12: Computed H-H chemical shifts (ppm) and J couplings (Hz) at the QM/FDE$_{\text{noMM}}$(3Å) level for MOED dissolved in aqueous solution. The atoms labeling is reported in fig. S1.

Atom	Shift	H(15)	H(16)	H(17)	H(18)	H(19)	H(20)	H(21)	H(22)	H(23)	H(24)	H(25)	H(26)	H(27)
H(15)	6.362	0.00	17.28	-0.02	-0.34	-0.37	-0.05	0.96	-1.27	0.33	-0.57	-0.52	-0.48	-0.18
H(16)	7.219	17.28	0.00	-1.00	-1.50	0.63	1.39	-0.19	-0.39	-0.50	-0.05	-0.01	-0.02	-0.05
H(17)	7.221	-0.02	-1.00	0.00	2.34	10.31	-0.17	-0.05	0.28	-0.20	-0.05	0.00	-0.01	-0.02
H(18)	7.720	-0.34	-1.50	2.34	0.00	-0.09	10.71	-0.16	-0.04	-0.12	0.27	0.00	0.00	-0.07
H(19)	6.723	-0.37	0.63	10.31	-0.09	0.00	1.69	0.13	0.10	0.23	-0.11	-0.48	-0.43	-0.07
H(20)	6.811	-0.05	1.39	-0.17	10.71	1.69	0.00	0.07	-0.01	0.21	0.01	-0.44	-0.40	-0.08
H(21)	6.993	0.96	-0.19	-0.05	-0.16	0.13	0.07	0.00	8.06	1.63	0.00	-0.94	-0.91	-0.82
H(22)	7.303	-1.27	-0.39	0.28	-0.04	0.10	-0.01	8.06	0.00	-0.00	2.19	0.05	0.05	-0.15
H(23)	6.978	0.33	-0.50	-0.20	-0.12	0.23	0.21	1.63	-0.00	0.00	8.19	-0.67	-0.67	-0.51
H(24)	6.930	-0.57	-0.05	-0.05	0.27	-0.11	0.01	0.00	2.19	8.19	0.00	-0.14	-0.16	-0.06
H(25)	3.607	-0.52	-0.01	0.00	0.00	-0.48	-0.44	-0.94	0.05	-0.67	-0.14	0.00	-23.50	-23.50
H(26)	3.556	-0.48	-0.02	-0.01	0.00	-0.43	-0.40	-0.91	0.05	-0.67	-0.16	-23.50	0.00	-23.55
H(27)	3.179	-0.18	-0.05	-0.02	-0.07	-0.07	-0.08	-0.82	-0.15	-0.51	-0.06	-23.50	-23.55	0.00

Table S13: Computed H-H chemical shifts (ppm) and J couplings (Hz) at the QM/FDE$_{\text{noMM}}$(3Å)/FQ^b level for MOED dissolved in aqueous solution. The atoms labeling is reported in fig. S1.

Atom	Shift	H(15)	H(16)	H(17)	H(18)	H(19)	H(20)	H(21)	H(22)	H(23)	H(24)	H(25)	H(26)	H(27)
H(15)	6.672	0.00	18.12	0.15	-0.21	-0.21	0.11	0.69	-1.40	0.07	-0.67	0.04	0.02	-0.18
H(16)	7.618	18.12	0.00	-0.88	-1.41	0.15	0.79	-0.08	-0.04	-0.29	0.18	-0.68	-0.62	-0.06
H(17)	7.446	0.15	-0.88	0.00	2.22	9.82	-0.08	-0.00	0.37	-0.12	0.00	-0.20	-0.19	-0.02
H(18)	7.983	-0.21	-1.41	2.22	0.00	0.02	10.12	-0.13	0.04	-0.04	0.31	-0.18	-0.16	-0.07
H(19)	6.587	-0.21	0.15	9.82	0.02	0.00	2.05	-0.03	0.03	-0.05	-0.09	-0.17	-0.16	-0.06
H(20)	6.661	0.11	0.79	-0.08	10.12	2.05	0.00	-0.08	-0.07	-0.02	0.03	-0.16	-0.15	-0.07
H(21)	7.760	0.69	-0.08	-0.00	-0.13	-0.03	-0.08	0.00	7.97	1.63	0.31	-1.49	-1.40	-0.77
H(22)	7.800	-1.40	-0.04	0.37	0.04	0.03	-0.07	7.97	0.00	0.42	2.04	0.37	0.34	-0.12
H(23)	7.778	0.07	-0.29	-0.12	-0.04	-0.05	-0.02	1.63	0.42	0.00	8.08	-1.27	-1.20	-0.57
H(24)	7.403	-0.67	0.18	0.00	0.31	-0.09	0.03	0.31	2.04	8.08	0.00	0.22	0.16	-0.01
H(25)	4.024	0.04	-0.68	-0.20	-0.18	-0.17	-0.16	-1.49	0.37	-1.27	0.22	0.00	-25.05	-24.09
H(26)	3.964	0.02	-0.62	-0.19	-0.16	-0.16	-0.15	-1.40	0.34	-1.20	0.16	-25.05	0.00	-23.99
H(27)	3.620	-0.18	-0.06	-0.02	-0.07	-0.06	-0.07	-0.77	-0.12	-0.57	-0.01	-24.09	23.99	0.00

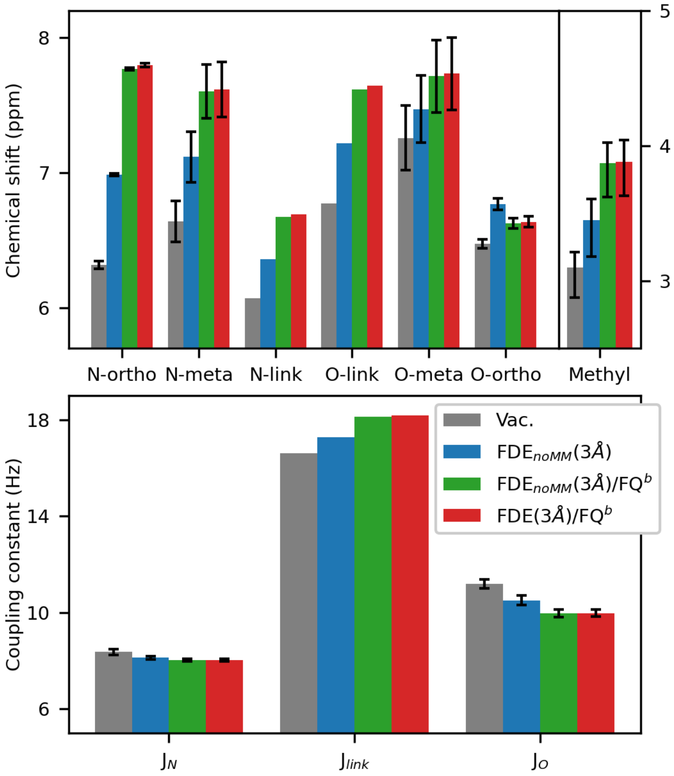

Figure S4: Computed QM/FDE$_{\text{noMM}}$(3Å), QM/FDE$_{\text{noMM}}$(3Å)/FQ^{b}, and QM/FDE(3Å)/FQ^{b} ^{1}H-NMR chemical shifts (ppm) and J coupling constants (Hz) of MOED dissolved in aqueous solution. Gas-phase (vac.) data are also reported. Error bars report the variation of the signals in the case of non-equivalent protons.

Hydrogen	Chemical shift (ppm)					Coupling Constant (Hz)				
	exp.	vac.	FQ^b	FDE/FQ^b	FDE/FQFμ	exp.	vac.	FQ^b	FDE/FQ^b	FDE/FQFμ
N-ortho	8.13	6.32 (±0.03)	8.34 (±0.03)	7.80 (±0.01)	7.91 (±0.02)	6.89	8.36 (±0.11)	8.04 (±0.05)	8.02 (±0.05)	8.07 (±0.05)
N-meta	7.65	6.64 (±0.15)	8.02 (±0.22)	7.62 (±0.20)	7.68 (±0.20)					
N-link	6.79	6.07	7.27	6.69	6.75	16.06	16.62	19.12	18.18	19.45
O-link	7.48	6.77	8.02	7.64	7.68					
O-meta	7.34	7.26 (±0.24)	8.14 (±0.29)	7.73 (±0.27)	7.77 (±0.27)	8.71	11.19 (±0.19)	9.98 (±0.11)	9.98 (±0.15)	9.95 (±0.10)
O-ortho	6.57	6.47 (±0.03)	6.95 (±0.04)	6.64 (±0.04)	6.62 (±0.04)					

Table S14: ^{1}H-NMR chemical shifts (ppm) and spin-spin couplings (Hz) data of MOED dissolved in aqueous solution. For each group of non-equivalent protons (see Fig. 1 of the main text for the definition) we report the experimental value (taken from Ref. 95 of the main text) and the computed values in gas-phase and within the QM/FQ^b, QM/FDE(3Å)/FQ^b, and QM/FDE(3Å)/FQFμ levels of theory for the case of aqueous solution. The half-difference between the equivalent protons is reported in parentheses. The atoms labeling is reported in fig. S1.

S2 Bond order analysis

As reported in the main text, we compute the delocalization indices (DI) of selected atom pairs (see Refs. 100 and 101 of the main text) of MOED in solution to investigate how the zwitterion-quinone equilibrium is affected by solvent effects. We focus on three portions of MOED: 1) the sum of DI of N-C bonds, in particular N-C(9), N-C(14), and N-C(11) ($\sum_{\mathrm{BO}} N$); C(8)-O; C(1)-C(2) bond between the rings. The atoms labeling is reported in fig. S1, and the numerical values of these indices are reported in table S15.

Table S15: Computed delocalization indices (DI) for three key portions of MOED: sum of N-C(9), N-C(14), and N-C(11) ($\sum_{\mathrm{BO}} N$, first column); C(8)-O DI (second column); C(1)-C(2) bond between the rings (third column). The atoms labeling is reported in fig. S1. DI are computed as defined in Refs. 100 and 101 of the main text. DI in solution are obtained by averaging the values obtained from 5 snapshots, and the maximum statistical error defined as (standard deviation)/(square root of number of frames) is 0.014. Computed gas phase DI calculations and theoretical values of zwitterion-quinone bond orders are reported for reference.

System	$\sum_{\mathrm{BO}} N$		C(8)-O		C(1)-C(2)	
Zwitterion	4.00		1.00		2.00	
Quinone	3.00		2.00		1.00	
Gas phase	3.17		1.40		1.37	
Solvent	FQ[b]	FDE/FQ[b]	FQ[b]	FDE/FQ[b]	FQ[b]	FDE/FQ[b]
THF	3.22	3.21	1.35	1.35	1.43	1.41
ACN	3.24	3.23	1.31	1.33	1.49	1.47
ETH	3.25	3.24	1.14	1.17	1.50	1.49
WAT	3.26	3.23	1.12	1.25	1.55	1.48

As stated in the main text, MOED is kept frozen during the MD simulation. Therefore, we expect that the bond order does not change significantly during the trajectory. To have a rough estimate of the fluctuations associated with the solvent configurations, in table S15 we compute the DI for five randomly selected snapshots of the MD simulation in each solvent, and we consider the averaged values of the DI. The statistical error, defined as the ratio between the standard deviation and the square root of the number of frames, reaches a maximum value of 0.014, which does not change significantly the interpretation of the numerical results.

By comparing DI to the gas phase, it can be noticed that the zwitterionic MOED is stabilized in solution independently of the nature of the solvent because the bond orders shift toward a higher zwitterionic character. Including the FDE layer, as opposed to a complete QM/FQ^b calculation, does not alter the electronic nature of MOED since the bond orders are almost the same between QM/FQ^b and QM/FDE/FQ^b. The most substantial impact of quantum confinement effects *via* the FDE layer inclusion is observed for the case of the aqueous solution, and a similar behavior comes out from the analysis of fig. 10 of the main text about ^{1}H-NMR chemical shifts and spin-spin coupling constants.

From the inspection of the numerical values in table S15, it can be stated that the zwitterion-quinone equilibrium is only slightly affected by the nature of the solvent since DI values only slightly vary. However, it is not straightforward to associate a zwitterionic or quinonoid form to MOED. In fact, the C(1)-C(2) DI carries a high contribution from the zwitterionic structure, also confirmed by the value of the J_{link}, which is compatible with two vinylic hydrogens. On the other hand, $\sum_{\mathrm{BO}} N$ has a more decisive contribution from the quinonoid form. The C(8)-O DI is more dependent on the nature of the solvent since polar protic solvents shift the bond order toward the zwitterionic nature, while THF and ACN present a higher contribution from the quinonoid character. The relatively small value of C(8)-O bond order, compatible with the zwitterion, is in contrast with the high value of chemical shift of C(8) of about 170-180 ppm compatible with a carbonylic carbon. Therefore, we can conclude that a more extended analysis would be needed to clarify the electronic properties of MOED.

Table S16: Computed C and H chemical shifts (ppm) in gas-phase and for each solvent as computed at the QM/FQ[b] and QM/FDE(3Å)/FQ[b] levels of theory. The value for each nucleus is accompanied by the difference with respect to the gas-phase calculation obtained as $\Delta = \delta_i - \delta_{vac}$ where δ_i and δ_{vac} are the chemical shift calculated with the indicated model and in gas-phase, respectively. Besides that, also the chemical shifts after the inclusion of the FDE shell (3 Å) are presented, together with the difference with respect to the values of the QM/FQ calculation as $\Delta\Delta = \delta_{i,FDE/MM} - \delta_{i,MM}$. The atoms labeling is reported in fig. S1.

Atom	Vac	Water		Ethanol		Acetonitrile		THF	
		Δ FQ[b]	ΔΔ FDE/FQ[b]	Δ FQ	ΔΔ FDE/FQ	Δ FQ	ΔΔ FDE/FQ	Δ FQ	ΔΔ FDE/FQ
H(15)	6.071	7.267 (1.196)	6.692 (-0.575)	6.885 (0.814)	6.722 (-0.162)	6.678 (0.607)	6.477 (-0.201)	6.3788 (0.307)	6.231 (-0.147)
H(16)	6.772	8.023 (1.251)	7.643 (-0.380)	7.893 (1.121)	7.750 (-0.143)	7.745 (0.973)	7.536 (-0.209)	7.4688 (0.696)	7.270 (-0.198)
H(17)	7.018	7.848 (0.830)	7.466 (-0.381)	7.631 (0.613)	7.522 (-0.109)	7.447 (0.429)	7.322 (-0.126)	7.2088 (0.190)	7.106 (-0.102)
H(18)	7.497	8.437 (0.940)	8.001 (-0.436)	8.190 (0.693)	8.058 (-0.132)	7.981 (0.484)	7.826 (-0.155)	7.8177 (0.320)	7.672 (-0.144)
H(19)	6.442	6.919 (0.477)	6.596 (-0.323)	6.856 (0.414)	6.793 (-0.063)	6.304 (-0.138)	6.322 (0.018)	6.1988 (-0.244)	6.208 (0.009)
H(20)	6.507	6.989 (0.482)	6.677 (-0.312)	6.892 (0.385)	6.839 (-0.053)	6.360 (-0.147)	6.386 (0.027)	6.2700 (-0.237)	6.286 (0.016)
H(21)	6.347	8.301 (1.954)	7.784 (-0.517)	7.917 (1.570)	7.789 (-0.128)	7.949 (1.602)	7.633 (-0.316)	7.6500 (1.303)	7.350 (-0.300)
H(22)	6.789	8.246 (1.457)	7.822 (-0.425)	7.964 (1.175)	7.839 (-0.126)	7.846 (1.057)	7.639 (-0.208)	7.5699 (0.780)	7.377 (-0.192)
H(23)	6.287	8.356 (2.069)	7.811 (-0.545)	8.000 (1.713)	7.848 (-0.152)	7.994 (1.707)	7.662 (-0.332)	7.6522 (1.365)	7.345 (-0.307)
H(24)	6.489	7.798 (1.309)	7.415 (-0.383)	7.484 (0.995)	7.361 (-0.124)	7.410 (0.921)	7.240 (-0.170)	7.1111 (0.622)	6.958 (-0.153)
H(25)	3.211	4.246 (1.035)	4.041 (-0.206)	4.060 (0.849)	4.048 (-0.012)	4.041 (0.830)	3.950 (-0.091)	3.8177 (0.606)	3.745 (-0.072)
H(26)	3.212	4.208 (0.996)	3.980 (-0.228)	4.054 (0.842)	4.020 (-0.035)	4.049 (0.837)	3.963 (-0.086)	3.8488 (0.636)	3.757 (-0.091)
H(27)	2.877	3.927 (1.050)	3.628 (-0.299)	3.723 (0.846)	3.666 (-0.058)	3.761 (0.884)	3.571 (-0.191)	3.6177 (0.740)	3.451 (-0.166)
C(1)	93.684	102.697 (9.013)	95.350 (-7.347)	98.511 (4.827)	96.988 (-1.522)	94.830 (1.146)	93.348 (-1.482)	91.986 (-1.698)	91.265 (-0.721)
C(2)	123.448	129.116 (5.668)	129.520 (0.404)	132.758 (9.310)	132.098 (-0.660)	129.708 (6.260)	129.218 (-0.. 490)	128.404 (4.956)	127.343 (-1.061)
C(3)	112.181	108.685 (-3.496)	104.390 (-4.295)	109.292 (-2.889)	108.651 (-0.641)	104.459 (-7.722)	104.782 (0.323)	104.801 (-7.380)	105.575 (0.774)
C(4)	124.951	124.323 (-0.628)	124.846 (0.523)	126.263 (1.312)	126.204 (-0.058)	125.454 (0.503)	125.494 (0.. 040)	125.506 (0.555)	125.352 (-0.154)
C(5)	114.863	115.564 (0.701)	115.329 (-0.235)	117.105 (2.242)	117.030 (-0.075)	115.962 (1.099)	115.872 (-00 .090)	116.157 (1.294)	115.919 (-0.238)
C(6)	113.984	105.108 (-8.876)	107.103 (1.995)	107.813 (-6.171)	108.037 (0.224)	106.788 (-7.196)	107.792 (11 .004)	108.456 (-5.528)	109.164 (0.708)
C(7)	115.499	106.936 (-8.563)	109.207 (2.271)	109.121 (-6.378)	109.458 (0.337)	108.866 (-6.633)	109.953 (11 .086)	110.751 (-4.748)	111.424 (0.674)
C(8)	181.078	169.987 (-11.091)	174.893 (4.907)	173.337 (-7.741)	174.077 (0.739)	175.706 (-5.372)	176.598 ((0.892)	176.860 (-4.218)	177.467 (0.607)
C(9)	121.624	130.263 (8.639)	127.983 (-2.280)	128.676 (7.052)	128.128 (-0.547)	129.150 (7.526)	127.864 (-11 .286)	127.637 (6.013)	126.325 (-1.312)
C(10)	100.391	104.443 (4.052)	102.273 (-2.169)	103.012 (2.621)	102.610 (-0.402)	101.500 (1.109)	101.418 (– 0.081)	99.847 (-0.544)	99.896 (0.049)
C(11)	119.387	131.520 (12.133)	128.429 (-3.091)	129.464 (10.077)	128.740 (-0.724)	129.909 (10.522)	128.0477 (-1.862)	127.916 (8.529)	126.063 (-1.853)
C(12)	109.496	111.609 (2.113)	109.600 (-2.010)	109.863 (0.367)	109.364 (-0.499)	108.731 (-0.765)	108.699 ((-0.032)	107.547 (-1.949)	107.693 (0.146)
C(13)	130.985	139.745 (8.760)	139.685 (-0.060)	140.434 (9.449)	140.253 (-0.181)	139.195 (8.210)	138.885 (– 0.311)	137.949 (6.964)	137.327 (-0.622)
C(14)	29.739	32.701 (2.962)	31.600 (-1.101)	31.879 (2.140)	31.711 (-0.168)	31.772 (2.033)	31.767 (-0.005)	30.769 (1.030)	30.539 (-0.230)

Table S17: Computed H-H J couplings (Hz) at the QM/FDE(3Å)/FQ[b] level for MOED dissolved in ethanol. The atoms labeling is reported in fig. S1.

Atom	H(15)	H(16)	H(17)	H(18)	H(19)	H(20)	H(21)	H(22)	H(23)	H(24)	H(25)	H(26)	H(27)
H(15)	0.00	17.88	0.28	-0.13	-0.32	-0.00	0.71	-1.45	0.09	-0.76	0.09	0.07	-0.17
H(16)	17.88	0.00	-0.95	-1.45	0.15	0.76	-0.08	-0.02	-0.28	0.22	-0.77	-0.71	-0.06
H(17)	0.28	-0.95	0.00	2.03	9.85	0.03	0.00	0.41	-0.11	0.03	-0.27	-0.25	-0.02
H(18)	-0.13	-1.45	2.03	0.00	0.17	10.18	-0.13	0.08	-0.03	0.35	-0.25	-0.23	-0.07
H(19)	-0.32	0.15	9.85	0.17	0.00	2.02	-0.03	-0.01	-0.07	-0.12	-0.09	-0.09	-0.05
H(20)	-0.00	0.76	0.03	10.18	2.02	0.00	-0.09	-0.11	-0.03	0.00	-0.09	-0.08	-0.07
H(21)	0.71	-0.08	0.00	-0.13	-0.03	-0.09	0.00	7.92	1.59	0.31	-1.50	-1.42	-0.80
H(22)	-1.45	-0.02	0.41	0.08	-0.01	-0.11	7.92	0.00	0.43	2.01	0.38	0.35	-0.12
H(23)	0.09	-0.28	-0.11	-0.03	-0.07	-0.03	1.59	0.43	0.00	8.09	-1.32	-1.26	-0.59
H(24)	-0.76	0.22	0.03	0.35	-0.12	0.00	0.31	2.01	8.09	0.00	0.23	0.17	-0.01
H(25)	0.09	-0.77	-0.27	-0.25	-0.09	-0.09	-1.50	0.38	-1.32	0.23	0.00	-25.45	-24.24
H(26)	0.07	-0.71	-0.25	-0.23	-0.09	-0.08	-1.42	0.35	-1.26	0.17	-25.45	0.00	-24.18
H(27)	-0.17	-0.06	-0.02	-0.07	-0.05	-0.07	-0.80	-0.12	-0.59	-0.01	-24.24	-24.18	0.00

Table S18: Computed H-H J couplings (Hz) at the QM/FDE(3Å)/FQ^b level for MOED dissolved in acetonitrile. The atoms labeling is reported in fig. S1.

Atom	H(15)	H(16)	H(17)	H(18)	H(19)	H(20)	H(21)	H(22)	H(23)	H(24)	H(25)	H(26)	H(27)
H(15)	0.00	17.52	0.04	-0.35	-0.18	0.16	0.71	-1.35	0.09	-0.66	-0.08	-0.08	-0.18
H(16)	17.52	0.00	-0.90	-1.39	0.22	0.90	-0.08	-0.16	-0.31	0.09	-0.51	-0.48	-0.05
H(17)	0.04	-0.90	0.00	2.16	9.78	-0.19	0.00	0.32	-0.12	-0.04	-0.14	-0.13	-0.02
H(18)	-0.35	-1.39	2.16	0.00	-0.10	10.08	-0.12	-0.00	-0.04	0.28	-0.12	-0.11	-0.07
H(19)	-0.18	0.22	9.78	-0.10	0.00	1.91	-0.01	0.06	-0.01	-0.08	-0.25	-0.23	-0.06
H(20)	0.16	0.90	-0.19	10.08	1.91	0.00	-0.06	-0.04	0.02	0.05	-0.24	-0.22	-0.07
H(21)	0.71	-0.08	0.00	-0.12	-0.01	-0.06	0.00	7.99	1.58	0.23	-1.37	-1.32	-0.77
H(22)	-1.35	-0.16	0.32	-0.00	0.06	-0.04	7.99	0.00	0.32	2.05	0.31	0.29	-0.12
H(23)	0.09	-0.31	-0.12	-0.04	-0.01	0.02	1.58	0.32	0.00	8.00	-1.16	-1.12	-0.54
H(24)	-0.66	0.09	-0.04	0.28	-0.08	0.05	0.23	2.05	8.00	0.00	0.14	0.10	-0.03
H(25)	-0.08	-0.51	-0.14	-0.12	-0.25	-0.24	-1.37	0.31	-1.16	0.14	0.00	-25.18	-23.90
H(26)	-0.08	-0.48	-0.13	-0.11	-0.23	-0.22	-1.32	0.29	-1.12	0.10	-25.18	0.00	-23.99
H(27)	-0.18	-0.05	-0.02	-0.07	-0.06	-0.07	-0.77	-0.12	-0.54	-0.03	-23.90	-23.99	0.00

Table S19: Computed H-H J couplings (Hz) at the QM/FDE(3Å)/FQ^b level for MOED dissolved in tetrahydrofuran. The atoms labeling is reported in fig. S1.

Atom	H(15)	H(16)	H(17)	H(18)	H(19)	H(20)	H(21)	H(22)	H(23)	H(24)	H(25)	H(26)	H(27)
H(15)	0.00	16.59	-0.04	-0.48	-0.21	0.13	0.84	-1.33	0.20	-0.62	-0.31	-0.29	-0.18
H(16)	16.59	0.00	-1.04	-1.49	0.46	1.22	-0.12	-0.35	-0.38	-0.02	-0.23	-0.22	-0.05
H(17)	-0.04	-1.04	0.00	2.13	10.04	-0.20	-0.02	0.29	-0.17	-0.06	-0.04	-0.05	-0.02
H(18)	-0.48	-1.49	2.13	0.00	-0.12	10.42	-0.14	-0.04	-0.09	0.27	-0.03	-0.03	-0.07
H(19)	-0.21	0.46	10.04	-0.12	0.00	1.57	0.04	0.11	0.09	-0.07	-0.39	-0.36	-0.07
H(20)	0.13	1.22	-0.20	10.42	1.57	0.00	-0.01	-0.00	0.09	0.05	-0.36	-0.34	-0.07
H(21)	0.84	-0.12	-0.02	-0.14	0.04	-0.01	0.00	8.17	1.65	0.09	-1.18	-1.14	-0.78
H(22)	-1.33	-0.35	0.29	-0.04	0.11	-0.00	8.17	0.00	0.15	2.18	0.19	0.18	-0.13
H(23)	0.20	-0.38	-0.17	-0.09	0.09	0.09	1.65	0.15	0.00	8.21	-0.93	-0.91	-0.57
H(24)	-0.62	-0.02	-0.06	0.27	-0.07	0.05	0.09	2.18	8.21	0.00	0.01	-0.03	-0.03
H(25)	-0.31	-0.23	-0.04	-0.03	-0.39	-0.36	-1.18	0.19	-0.93	0.01	0.00	-24.40	-24.08
H(26)	-0.29	-0.22	-0.05	-0.03	-0.36	-0.34	-1.14	0.18	-0.91	-0.03	-24.40	0.00	-24.18
H(27)	-0.18	-0.05	-0.02	-0.07	-0.07	-0.07	-0.78	-0.13	-0.57	-0.03	-24.08	-24.18	0.00

Table S20: Computed H-H J couplings (Hz) at the QM/FQ^b level for MOED dissolved in ethanol. The atoms labeling is reported in fig. S1.

Atom	H(15)	H(16)	H(17)	H(18)	H(19)	H(20)	H(21)	H(22)	H(23)	H(24)	H(25)	H(26)	H(27)
H(15)	0.00	17.92	0.32	-0.11	-0.34	-0.02	0.70	-1.48	0.09	-0.82	0.16	0.13	-0.17
H(16)	17.92	0.00	-1.03	-1.47	0.14	0.74	-0.09	0.01	-0.28	0.27	-0.86	-0.78	-0.06
H(17)	0.32	-1.03	0.00	1.98	9.87	0.08	-0.00	0.42	-0.12	0.05	-0.29	-0.27	-0.02
H(18)	-0.11	-1.47	1.98	0.00	0.22	10.17	-0.13	0.10	-0.03	0.36	-0.27	-0.25	-0.07
H(19)	-0.34	0.14	9.87	0.22	0.00	2.05	-0.04	-0.03	-0.08	-0.13	-0.06	-0.07	-0.05
H(20)	-0.02	0.74	0.08	10.17	2.05	0.00	-0.09	-0.12	-0.04	-0.01	-0.06	-0.06	-0.07
H(21)	0.70	-0.09	-0.00	-0.13	-0.04	-0.09	0.00	7.95	1.58	0.36	-1.57	-1.49	-0.82
H(22)	-1.48	0.01	0.42	0.10	-0.03	-0.12	7.95	0.00	0.48	1.99	0.43	0.40	-0.11
H(23)	0.09	-0.28	-0.12	-0.03	-0.08	-0.04	1.58	0.48	0.00	8.12	-1.41	-1.34	-0.61
H(24)	-0.82	0.27	0.05	0.36	-0.13	-0.01	0.36	1.99	8.12	0.00	0.28	0.22	-0.00
H(25)	0.16	-0.86	-0.29	-0.27	-0.06	-0.06	-1.57	0.43	-1.41	0.28	0.00	-26.06	-24.29
H(26)	0.13	-0.78	-0.27	-0.25	-0.07	-0.06	-1.49	0.40	-1.34	0.22	-26.06	0.00	-24.22
H(27)	-0.17	-0.06	-0.02	-0.07	-0.05	-0.07	-0.82	-0.11	-0.61	-0.00	-24.29	-24.22	0.00

Table S21: Computed H-H J couplings (Hz) at the QM/FQ^b level for MOED dissolved in acetonitrile. The atoms labeling is reported in fig. S1.

Atom	H(15)	H(16)	H(17)	H(18)	H(19)	H(20)	H(21)	H(22)	H(23)	H(24)	H(25)	H(26)	H(27)
H(15)	0.00	17.77	0.10	-0.32	-0.18	0.17	0.67	-1.41	0.06	-0.75	0.06	0.05	-0.18
H(16)	17.77	0.00	-0.94	-1.40	0.15	0.79	-0.09	-0.07	-0.30	0.17	-0.69	-0.65	-0.05
H(17)	0.10	-0.94	0.00	2.10	9.69	-0.14	-0.00	0.35	-0.12	-0.01	-0.18	-0.17	-0.02
H(18)	-0.32	-1.40	2.10	0.00	-0.04	9.95	-0.13	0.02	-0.04	0.30	-0.16	-0.15	-0.07
H(19)	-0.18	0.15	9.69	-0.04	0.00	2.00	-0.03	0.05	-0.05	-0.08	-0.19	-0.18	-0.06
H(20)	0.17	0.79	-0.14	9.95	2.00	0.00	-0.08	-0.05	-0.02	0.05	-0.19	-0.18	-0.07
H(21)	0.67	-0.09	-0.00	-0.13	-0.03	-0.08	0.00	8.04	1.55	0.34	-1.56	-1.50	-0.79
H(22)	-1.41	-0.07	0.35	0.02	0.05	-0.05	8.04	0.00	0.45	2.03	0.43	0.40	-0.11
H(23)	0.06	-0.30	-0.12	-0.04	-0.05	-0.02	1.55	0.45	0.00	8.04	-1.38	-1.33	-0.61
H(24)	-0.75	0.17	-0.01	0.30	-0.08	0.05	0.34	2.03	8.04	0.00	0.26	0.21	0.00
H(25)	0.06	-0.69	-0.18	-0.16	-0.19	-0.19	-1.56	0.43	-1.38	0.26	0.00	-26.44	-24.31
H(26)	0.05	-0.65	-0.17	-0.15	-0.18	-0.18	-1.50	0.40	-1.33	0.21	-26.44	0.00	-24.30
H(27)	-0.18	-0.05	-0.02	-0.07	-0.06	-0.07	-0.79	-0.11	-0.61	0.00	-24.31	-24.30	0.00

Table S22: Computed H-H J couplings (Hz) at the QM/FQ^b level for MOED dissolved in tetrahydrofuran. The atoms labeling is reported in fig. S1.

Atom	H(15)	H(16)	H(17)	H(18)	H(19)	H(20)	H(21)	H(22)	H(23)	H(24)	H(25)	H(26)	H(27)
H(15)	0.00	16.60	-0.02	-0.48	-0.17	0.18	0.78	-1.36	0.14	-0.69	-0.19	-0.18	-0.18
H(16)	16.60	0.00	-1.03	-1.44	0.33	1.06	-0.10	-0.30	-0.34	0.03	-0.39	-0.37	-0.05
H(17)	-0.02	-1.03	0.00	2.12	9.99	-0.22	-0.01	0.31	-0.14	-0.05	-0.10	-0.09	-0.02
H(18)	-0.48	-1.44	2.12	0.00	-0.14	10.35	-0.13	-0.02	-0.06	0.27	-0.09	-0.08	-0.07
H(19)	-0.17	0.33	9.99	-0.14	0.00	1.65	0.01	0.10	0.03	-0.06	-0.33	-0.31	-0.06
H(20)	0.18	1.06	-0.22	10.35	1.65	0.00	-0.05	-0.01	0.05	0.06	-0.31	-0.28	-0.07
H(21)	0.78	-0.10	-0.01	-0.13	0.01	-0.05	0.00	8.19	1.64	0.16	-1.34	-1.29	-0.81
H(22)	-1.36	-0.30	0.31	-0.02	0.10	-0.01	8.19	0.00	0.25	2.15	0.28	0.27	-0.12
H(23)	0.14	-0.34	-0.14	-0.06	0.03	0.05	1.64	0.25	0.00	8.22	-1.13	-1.09	-0.62
H(24)	-0.69	0.03	-0.05	0.27	-0.06	0.06	0.16	2.15	8.22	0.00	0.12	0.07	-0.01
H(25)	-0.19	-0.39	-0.10	-0.09	-0.33	-0.31	-1.34	0.28	-1.13	0.12	0.00	-25.42	-24.38
H(26)	-0.18	-0.37	-0.09	-0.08	-0.31	-0.28	-1.29	0.27	-1.09	0.07	-25.42	0.00	-24.47
H(27)	-0.18	-0.05	-0.02	-0.07	-0.06	-0.07	-0.81	-0.12	-0.62	-0.01	-24.38	-24.47	0.00

Table S23: Computed C-C J couplings (Hz) at the QM level for MOED in gas-phase. The atoms labeling is reported in fig. S1.

Atom	C(1)	C(2)	C(3)	C(4)	C(5)	C(6)	C(7)	C(8)	C(9)	C(10)	C(11)	C(12)	C(13)	C(14)
C(1)	0.00	60.98	-1.46	6.08	5.07	-0.88	-1.37	2.50	3.50	0.00	5.01	2.70	58.48	0.00
C(2)	60.98	0.00	58.57	1.92	0.00	6.53	6.23	-3.02	0.00	4.76	0.00	6.05	-1.10	0.52
C(3)	-1.46	58.57	0.00	48.53	47.68	-2.37	-2.28	8.96	0.00	-0.97	1.13	-1.38	8.94	0.00
C(4)	6.08	1.92	48.53	0.00	2.30	49.57	1.08	-0.86	0.00	0.68	0.00	1.17	-1.98	0.00
C(5)	5.07	0.00	47.68	2.30	0.00	0.92	50.08	-0.88	0.00	0.87	0.00	0.99	-1.88	0.00
C(6)	-0.88	6.53	-2.37	49.57	0.92	0.00	14.53	47.12	0.00	0.00	0.82	-0.54	2.03	0.00
C(7)	-1.37	6.23	-2.28	1.08	50.08	14.53	0.00	46.10	0.00	0.00	0.75	-0.51	1.88	0.00
C(8)	2.50	-3.02	8.96	-0.86	-0.88	47.12	46.10	0.00	0.00	1.03	0.00	1.42	-1.84	0.57
C(9)	3.50	0.00	0.00	0.00	0.00	0.00	0.00	0.00	0.00	58.28	0.00	4.62	-1.37	2.28
C(10)	0.00	4.76	-0.97	0.68	0.87	0.00	0.00	1.03	58.28	0.00	4.28	2.15	46.73	1.75
C(11)	5.01	0.00	1.13	0.00	0.00	0.82	0.75	0.00	0.00	4.28	0.00	58.44	-1.97	2.33
C(12)	2.70	6.05	-1.38	1.17	0.99	-0.54	-0.51	1.42	4.62	2.15	58.44	0.00	45.10	1.59
C(13)	58.48	-1.10	8.94	-1.98	-1.88	2.03	1.88	-1.84	-1.37	46.73	-1.97	45.10	0.00	0.00
C(14)	0.00	0.52	0.00	0.00	0.00	0.00	0.00	0.57	2.28	1.75	2.33	1.59	0.00	0.00

Table S24: Computed C-C J couplings (Hz) at the QM/FDE(3Å)/FQ^{b} level for MOED dissolved in aqueous solution. The atoms labeling is reported in fig. S1.

Atom	C(1)	C(2)	C(3)	C(4)	C(5)	C(6)	C(7)	C(8)	C(9)	C(10)	C(11)	C(12)	C(13)	C(14)
C(1)	0.00	58.88	1.23	4.70	3.56	0.44	-0.21	0.40	3.18	1.23	4.17	4.05	53.94	0.30
C(2)	58.88	0.00	53.62	4.28	2.49	4.37	3.90	-0.59	-0.07	4.22	0.31	4.99	1.93	-0.05
C(3)	1.23	53.62	0.00	46.17	44.93	-0.79	-0.81	6.51	0.17	-0.27	0.27	-0.02	5.55	0.06
C(4)	4.70	4.28	46.17	0.00	2.83	48.97	1.49	1.04	-0.01	0.11	0.00	0.22	-0.26	0.01
C(5)	3.56	2.49	44.93	2.83	0.00	1.37	49.83	1.00	0.02	0.32	-0.03	0.11	-0.31	0.01
C(6)	0.44	4.37	-0.79	48.97	1.37	0.00	12.95	45.54	0.09	-0.03	0.11	-0.02	0.27	0.03
C(7)	-0.21	3.90	-0.81	1.49	49.83	12.95	0.00	44.74	0.07	0.05	0.10	-0.03	0.21	0.03
C(8)	0.40	-0.59	6.51	1.04	1.00	45.54	44.74	0.00	0.05	0.14	0.05	0.14	0.05	0.04
C(9)	3.18	-0.07	0.17	-0.01	0.02	0.09	0.07	0.05	0.00	56.97	-0.51	5.67	-1.26	2.19
C(10)	1.23	4.22	-0.27	0.11	0.32	-0.03	0.05	0.14	56.97	0.00	5.46	0.98	44.65	1.90
C(11)	4.17	0.31	0.27	0.00	-0.03	0.11	0.10	0.05	-0.51	5.46	0.00	56.59	-1.28	2.11
C(12)	4.05	4.99	-0.02	0.22	0.11	-0.02	-0.03	0.14	5.67	0.98	56.59	0.00	43.40	1.81
C(13)	53.94	1.93	5.55	-0.26	-0.31	0.27	0.21	0.05	-1.26	44.65	-1.28	43.40	0.00	-0.45
C(14)	0.30	-0.05	0.06	0.01	0.01	0.03	0.03	0.04	2.19	1.90	2.11	1.81	-0.45	0.00

Table S25: Computed C-C J couplings (Hz) at the QM/FDE(3Å)/FQ^{b} level for MOED dissolved in ethanol. The atoms labeling is reported in fig. S1.

Atom	C(1)	C(2)	C(3)	C(4)	C(5)	C(6)	C(7)	C(8)	C(9)	C(10)	C(11)	C(12)	C(13)	C(14)
C(1)	0.00	59.22	1.05	4.91	3.82	0.31	-0.30	0.62	3.19	1.19	4.16	3.98	54.44	0.30
C(2)	59.22	0.00	54.54	4.28	2.42	4.48	3.94	-0.76	-0.08	4.28	0.28	5.03	1.91	-0.06
C(3)	1.05	54.54	0.00	46.98	45.81	-1.08	-1.05	7.14	0.21	-0.29	0.30	-0.04	5.52	0.09
C(4)	4.91	4.28	46.98	0.00	2.51	51.70	2.10	1.02	-0.04	0.14	-0.01	0.24	-0.29	-0.00
C(5)	3.82	2.42	45.81	2.51	0.00	2.02	52.89	1.03	-0.01	0.35	-0.05	0.14	-0.32	-0.00
C(6)	0.31	4.48	-1.08	51.70	2.02	0.00	10.63	46.22	0.12	-0.06	0.13	-0.04	0.31	0.05
C(7)	-0.30	3.94	-1.05	2.10	52.89	10.63	0.00	46.24	0.11	0.02	0.12	-0.05	0.24	0.05
C(8)	0.62	-0.76	7.14	1.02	1.03	46.22	46.24	0.00	-0.01	0.21	0.01	0.18	-0.00	0.01
C(9)	3.19	-0.08	0.21	-0.04	-0.01	0.12	0.11	-0.01	0.00	56.71	-0.51	5.62	-1.27	2.17
C(10)	1.19	4.28	-0.29	0.14	0.35	-0.06	0.02	0.21	56.71	0.00	5.42	1.05	44.56	1.89
C(11)	4.16	0.28	0.30	-0.01	-0.05	0.13	0.12	0.01	-0.51	5.42	0.00	56.60	-1.30	2.10
C(12)	3.98	5.03	-0.04	0.24	0.14	-0.04	-0.05	0.18	5.62	1.05	56.60	0.00	43.17	1.81
C(13)	54.44	1.91	5.52	-0.29	-0.32	0.31	0.24	-0.00	-1.27	44.56	-1.30	43.17	0.00	-0.45
C(14)	0.30	-0.06	0.09	-0.00	-0.00	0.05	0.05	0.01	2.17	1.89	2.10	1.81	-0.45	0.00

Table S26: Computed C-C J couplings (Hz) at the QM/FDE(3Å)/FQ^{b} level for MOED dissolved in acetonitrile. The atoms labeling is reported in fig. S1.

Atom	C(1)	C(2)	C(3)	C(4)	C(5)	C(6)	C(7)	C(8)	C(9)	C(10)	C(11)	C(12)	C(13)	C(14)
C(1)	0.00	58.81	1.26	4.57	3.48	0.49	-0.16	0.33	3.16	1.25	4.17	4.04	54.41	0.25
C(2)	58.81	0.00	53.59	4.20	2.44	4.41	4.03	-0.56	0.01	4.13	0.37	4.93	1.96	-0.01
C(3)	1.26	53.59	0.00	47.11	45.77	-0.68	-0.72	6.34	0.12	-0.23	0.25	-0.01	5.63	0.02
C(4)	4.57	4.20	47.11	0.00	3.19	48.63	0.89	0.94	0.03	0.09	0.02	0.23	-0.27	0.04
C(5)	3.48	2.44	45.77	3.19	0.00	0.77	49.18	0.88	0.06	0.30	-0.01	0.12	-0.31	0.04
C(6)	0.49	4.41	-0.68	48.63	0.77	0.00	15.31	45.83	0.08	0.01	0.12	-0.00	0.29	0.01
C(7)	-0.16	4.03	-0.72	0.89	49.18	15.31	0.00	45.08	0.06	0.09	0.11	-0.01	0.24	0.01
C(8)	0.33	-0.56	6.34	0.94	0.88	45.83	45.08	0.00	0.11	0.13	0.09	0.16	0.05	0.09
C(9)	3.16	0.01	0.12	0.03	0.06	0.08	0.06	0.11	0.00	56.96	-0.37	5.49	-1.19	2.19
C(10)	1.25	4.13	-0.23	0.09	0.30	0.01	0.09	0.13	56.96	0.00	5.27	1.22	44.66	1.88
C(11)	4.17	0.37	0.25	0.02	-0.01	0.12	0.11	0.09	-0.37	5.27	0.00	56.62	-1.24	2.14
C(12)	4.04	4.93	-0.01	0.23	0.12	-0.00	-0.01	0.16	5.49	1.22	56.62	0.00	43.42	1.80
C(13)	54.41	1.96	5.63	-0.27	-0.31	0.29	0.24	0.05	-1.19	44.66	-1.24	43.42	0.00	-0.41
C(14)	0.25	-0.01	0.02	0.04	0.04	0.01	0.01	0.09	2.19	1.88	2.14	1.80	-0.41	0.00

Table S27: Computed C-C J couplings (Hz) at the QM/FDE(3Å)/FQ^{b} level for MOED dissolved in tetrahydrofuran. The atoms labeling is reported in fig. S1.

Atom	C(1)	C(2)	C(3)	C(4)	C(5)	C(6)	C(7)	C(8)	C(9)	C(10)	C(11)	C(12)	C(13)	C(14)
C(1)	0.00	57.79	1.05	4.68	3.69	0.45	-0.19	0.49	3.13	1.20	4.15	3.94	56.22	0.15
C(2)	57.79	0.00	55.59	3.87	2.22	4.60	4.33	-0.77	0.11	4.19	0.39	4.98	1.78	0.07
C(3)	1.05	55.59	0.00	47.01	46.24	-0.82	-0.85	6.77	0.08	-0.20	0.28	-0.08	5.82	-0.06
C(4)	4.68	3.87	47.01	0.00	3.37	49.11	0.54	0.73	0.07	0.12	0.03	0.31	-0.38	0.09
C(5)	3.69	2.22	46.24	3.37	0.00	0.36	49.84	0.65	0.11	0.33	-0.01	0.18	-0.38	0.09
C(6)	0.45	4.60	-0.82	49.11	0.36	0.00	15.96	45.95	0.08	0.03	0.18	-0.02	0.42	-0.01
C(7)	-0.19	4.33	-0.85	0.54	49.84	15.96	0.00	44.97	0.06	0.11	0.16	-0.04	0.38	-0.01
C(8)	0.49	-0.77	6.77	0.73	0.65	45.95	44.97	0.00	0.18	0.19	0.11	0.26	-0.08	0.16
C(9)	3.13	0.11	0.08	0.07	0.11	0.08	0.06	0.18	0.00	57.66	-0.11	5.18	-1.14	2.21
C(10)	1.20	4.19	-0.20	0.12	0.33	0.03	0.11	0.19	57.66	0.00	4.90	1.60	44.42	1.87
C(11)	4.15	0.39	0.28	0.03	-0.01	0.18	0.16	0.11	-0.11	4.90	0.00	57.53	-1.23	2.17
C(12)	3.94	4.98	-0.08	0.31	0.18	-0.02	-0.04	0.26	5.18	1.60	57.53	0.00	42.70	1.77
C(13)	56.22	1.78	5.82	-0.38	-0.38	0.42	0.38	-0.08	-1.14	44.42	-1.23	42.70	0.00	-0.32
C(14)	0.15	0.07	-0.06	0.09	0.09	-0.01	-0.01	0.16	2.21	1.87	2.17	1.77	-0.32	0.00

Table S28: Computed C-C J couplings (Hz) at the QM/FQ^b level for MOED dissolved in aqueous solution. The atoms labeling is reported in fig. S1.

Atom	C(1)	C(2)	C(3)	C(4)	C(5)	C(6)	C(7)	C(8)	C(9)	C(10)	C(11)	C(12)	C(13)	C(14)
C(1)	0.00	59.01	0.90	5.24	4.02	0.12	-0.51	0.84	3.28	1.12	4.25	4.04	54.24	0.40
C(2)	59.01	0.00	54.43	4.29	2.13	4.65	3.95	-1.12	-0.33	4.63	0.07	5.41	1.61	-0.13
C(3)	0.90	54.43	0.00	45.30	43.68	-1.19	-1.22	7.45	0.33	-0.45	0.41	-0.17	5.76	0.14
C(4)	5.24	4.29	45.30	0.00	2.13	50.23	2.56	0.69	-0.11	0.19	-0.08	0.28	-0.41	-0.03
C(5)	4.02	2.13	43.68	2.13	0.00	2.45	51.08	0.71	-0.08	0.40	-0.12	0.18	-0.49	-0.03
C(6)	0.12	4.65	-1.19	50.23	2.45	0.00	10.10	45.44	0.14	-0.13	0.14	-0.10	0.42	0.05
C(7)	-0.51	3.95	-1.22	2.56	51.08	10.10	0.00	44.86	0.14	-0.05	0.13	-0.11	0.32	0.05
C(8)	0.84	-1.12	7.45	0.69	0.71	45.44	44.86	0.00	-0.09	0.24	-0.07	0.22	-0.18	-0.01
C(9)	3.28	-0.33	0.33	-0.11	-0.08	0.14	0.14	-0.09	0.00	55.96	-0.92	6.04	-1.53	2.18
C(10)	1.12	4.63	-0.45	0.19	0.40	-0.13	-0.05	0.24	55.96	0.00	5.89	0.40	43.89	1.90
C(11)	4.25	0.07	0.41	-0.08	-0.12	0.14	0.13	-0.07	-0.92	5.89	0.00	55.60	-1.51	2.03
C(12)	4.04	5.41	-0.17	0.28	0.18	-0.10	-0.11	0.22	6.04	0.40	55.60	0.00	42.79	1.82
C(13)	54.24	1.61	5.76	-0.41	-0.49	0.42	0.32	-0.18	-1.53	43.89	-1.51	42.79	0.00	-0.55
C(14)	0.40	-0.13	0.14	-0.03	-0.03	0.05	0.05	-0.01	2.18	1.90	2.03	1.82	-0.55	0.00

Table S29: Computed C-C J couplings (Hz) at the QM/FQ^b level for MOED dissolved in ethanol. The atoms labeling is reported in fig. S1.

Atom	C(1)	C(2)	C(3)	C(4)	C(5)	C(6)	C(7)	C(8)	C(9)	C(10)	C(11)	C(12)	C(13)	C(14)
C(1)	0.00	59.04	1.07	5.02	3.90	0.28	-0.35	0.67	3.20	1.19	4.18	4.04	54.24	0.32
C(2)	59.04	0.00	54.54	4.36	2.39	4.54	3.96	-0.85	-0.13	4.36	0.25	5.12	1.95	-0.08
C(3)	1.07	54.54	0.00	46.57	45.28	-1.11	-1.09	7.32	0.24	-0.32	0.32	-0.06	5.58	0.10
C(4)	5.02	4.36	46.57	0.00	2.41	51.41	2.26	0.96	-0.06	0.15	-0.03	0.25	-0.31	-0.01
C(5)	3.90	2.39	45.28	2.41	0.00	2.18	52.50	0.97	-0.02	0.36	-0.06	0.14	-0.35	-0.01
C(6)	0.28	4.54	-1.11	51.41	2.18	0.00	10.34	46.21	0.13	-0.08	0.13	-0.05	0.32	0.05
C(7)	-0.35	3.96	-1.09	2.26	52.50	10.34	0.00	46.18	0.11	0.00	0.12	-0.06	0.25	0.05
C(8)	0.67	-0.85	7.32	0.96	0.97	46.21	46.18	0.00	-0.03	0.22	-0.01	0.19	-0.04	-0.00
C(9)	3.20	-0.13	0.24	-0.06	-0.02	0.13	0.11	-0.03	0.00	56.10	-0.60	5.71	-1.31	2.18
C(10)	1.19	4.36	-0.32	0.15	0.36	-0.08	0.00	0.22	56.10	0.00	5.51	0.96	44.11	1.89
C(11)	4.18	0.25	0.32	-0.03	-0.06	0.13	0.12	-0.01	-0.60	5.51	0.00	55.91	-1.32	2.09
C(12)	4.04	5.12	-0.06	0.25	0.14	-0.05	-0.06	0.19	5.71	0.96	55.91	0.00	42.82	1.81
C(13)	54.24	1.95	5.58	-0.31	-0.35	0.32	0.25	-0.04	-1.31	44.11	-1.32	42.82	0.00	-0.48
C(14)	0.32	-0.08	0.10	-0.01	-0.01	0.05	0.05	-0.00	2.18	1.89	2.09	1.81	-0.48	0.00

Table S30: Computed C-C J couplings (Hz) at the QM/FQ^{b} level for MOED dissolved in acetonitrile. The atoms labeling is reported in fig. S1.

Atom	C(1)	C(2)	C(3)	C(4)	C(5)	C(6)	C(7)	C(8)	C(9)	C(10)	C(11)	C(12)	C(13)	C(14)
C(1)	0.00	58.38	1.32	4.67	3.53	0.49	-0.17	0.28	3.15	1.27	4.14	4.13	54.13	0.29
C(2)	58.38	0.00	53.49	4.35	2.44	4.39	3.93	-0.52	-0.07	4.24	0.31	5.04	2.02	-0.05
C(3)	1.32	53.49	0.00	46.61	45.09	-0.64	-0.67	6.33	0.15	-0.26	0.26	-0.01	5.61	0.05
C(4)	4.67	4.35	46.61	0.00	3.16	48.08	0.97	0.96	-0.00	0.09	0.01	0.22	-0.25	0.02
C(5)	3.53	2.44	45.09	3.16	0.00	0.87	48.49	0.91	0.04	0.30	-0.03	0.11	-0.30	0.02
C(6)	0.49	4.39	-0.64	48.08	0.87	0.00	15.52	45.66	0.07	-0.01	0.10	-0.00	0.26	0.02
C(7)	-0.17	3.93	-0.67	0.97	48.49	15.52	0.00	44.87	0.06	0.07	0.09	-0.01	0.19	0.02
C(8)	0.28	-0.52	6.33	0.96	0.91	45.66	44.87	0.00	0.08	0.10	0.07	0.12	0.08	0.06
C(9)	3.15	-0.07	0.15	-0.00	0.04	0.07	0.06	0.08	0.00	55.88	-0.60	5.68	-1.29	2.19
C(10)	1.27	4.24	-0.26	0.09	0.30	-0.01	0.07	0.10	55.88	0.00	5.47	0.98	43.83	1.87
C(11)	4.14	0.31	0.26	0.01	-0.03	0.10	0.09	0.07	-0.60	5.47	0.00	55.61	-1.30	2.09
C(12)	4.13	5.04	-0.01	0.22	0.11	-0.00	-0.01	0.12	5.68	0.98	55.61	0.00	42.75	1.79
C(13)	54.13	2.02	5.61	-0.25	-0.30	0.26	0.19	0.08	-1.29	43.83	-1.30	42.75	0.00	-0.46
C(14)	0.29	-0.05	0.05	0.02	0.02	0.02	0.02	0.06	2.19	1.87	2.09	1.79	-0.46	0.00

Table S31: Computed C-C J couplings (Hz) at the QM/FQ^{b} level for MOED dissolved in tetrahydrofuran. The atoms labeling is reported in fig. S1.

Atom	C(1)	C(2)	C(3)	C(4)	C(5)	C(6)	C(7)	C(8)	C(9)	C(10)	C(11)	C(12)	C(13)	C(14)
C(1)	0.00	57.27	1.23	4.67	3.65	0.52	-0.14	0.36	3.11	1.26	4.09	4.08	55.78	0.20
C(2)	57.27	0.00	55.30	4.11	2.36	4.49	4.17	-0.63	0.08	4.24	0.39	5.01	1.99	0.03
C(3)	1.23	55.30	0.00	46.50	45.60	-0.70	-0.73	6.66	0.08	-0.19	0.24	-0.01	5.66	-0.02
C(4)	4.67	4.11	46.50	0.00	3.42	48.54	0.53	0.82	0.05	0.09	0.03	0.26	-0.30	0.07
C(5)	3.65	2.36	45.60	3.42	0.00	0.37	49.13	0.75	0.09	0.30	-0.00	0.13	-0.31	0.06
C(6)	0.52	4.49	-0.70	48.54	0.37	0.00	16.30	45.78	0.07	0.03	0.14	0.00	0.33	0.00
C(7)	-0.14	4.17	-0.73	0.53	49.13	16.30	0.00	44.78	0.05	0.11	0.12	-0.01	0.29	0.00
C(8)	0.36	-0.63	6.66	0.82	0.75	45.78	44.78	0.00	0.15	0.14	0.11	0.19	0.02	0.12
C(9)	3.11	0.08	0.08	0.05	0.09	0.07	0.05	0.15	0.00	56.65	-0.32	5.34	-1.19	2.23
C(10)	1.26	4.24	-0.19	0.09	0.30	0.03	0.11	0.14	56.65	0.00	5.07	1.41	43.56	1.87
C(11)	4.09	0.39	0.24	0.03	-0.00	0.14	0.12	0.11	-0.32	5.07	0.00	56.63	-1.24	2.14
C(12)	4.08	5.01	-0.01	0.26	0.13	0.00	-0.01	0.19	5.34	1.41	56.63	0.00	42.02	1.77
C(13)	55.78	1.99	5.66	-0.30	-0.31	0.33	0.29	0.02	-1.19	43.56	-1.24	42.02	0.00	-0.37
C(14)	0.20	0.03	-0.02	0.07	0.06	0.00	0.00	0.12	2.23	1.87	2.14	1.77	-0.37	0.00
